# Performance and Deployment of Low-Cost Particle Sensor Units to Monitor Biomass Burning Events and Their Application in an Educational Initiative

**DOI:** 10.3390/s21217206

**Published:** 2021-10-29

**Authors:** Fabienne Reisen, Jacinta Cooper, Jennifer C. Powell, Christopher Roulston, Amanda J. Wheeler

**Affiliations:** 1 CSIRO Oceans & Atmosphere, Private Bag 1, Aspendale, VIC 3195, Australia; jacintacooper29@gmail.com (J.C.); jennifer.powell@csiro.au (J.C.P.); Chris.roulston@csiro.au (C.R.); 2 Mary MacKillop Institute for Health Research, Australian Catholic University, Melbourne, VIC 3000, Australia; Amanda.Wheeler@acu.edu.au; 3 Menzies Institute for Medical Research, University of Tasmania, Hobart, TAS 7000, Australia

**Keywords:** particulate matter, validation, smoke, sensors, STEM, air quality

## Abstract

Biomass burning smoke is often a significant source of airborne fine particles in regional areas where air quality monitoring is scarce. Emerging sensor technology provides opportunities to monitor air quality on a much larger geographical scale with much finer spatial resolution. It can also engage communities in the conversation around local pollution sources. The SMoke Observation Gadget (SMOG), a unit with a Plantower dust sensor PMS3003, was designed as part of a school-based Science, Technology, Engineering and Mathematics (STEM) project looking at smoke impacts in regional areas of Victoria, Australia. A smoke-specific calibration curve between the SMOG units and a standard regulatory instrument was developed using an hourly data set collected during a peat fire. The calibration curve was applied to the SMOG units during all field-based validation measurements at several locations and during different seasons. The results showed strong associations between individual SMOG units for PM_2.5_ concentrations (r^2^ = 0.93–0.99) and good accuracy (mean absolute error (MAE) < 2 μg m^−3^). Correlations of the SMOG units to reference instruments also demonstrated strong associations (r^2^ = 0.87–95) and good accuracy (MAE of 2.5–3.0 μg m^−3^). The PM_2.5_ concentrations tracked by the SMOG units had a similar response time as those measured by collocated reference instruments. Overall, the study has shown that the SMOG units provide relevant information about ambient PM_2.5_ concentrations in an airshed impacted predominantly by biomass burning, provided that an adequate adjustment factor is applied.

## 1. Introduction

It is well understood that there are negative health impacts from exposures to biomass smoke [1,2,3,4,5,6,7,8,9,10]. With the increasing amount of time when bushfires and prescribed burns can occur, there is growing interest from the public on how to best measure and manage their exposures [11,12,13,14].

To capture the local movement of smoke associated with biomass burning events a dense spatial network of air quality monitors is needed [15,16], which can be enhanced through the use of satellite-based data [15,17,18]. Considerable resources are required to maintain such a network of reference air quality monitors in regional locations where biomass smoke events most frequently occur [19]. In response to this resourcing issue, the use of ‘low-cost’ air quality sensors can provide a useful alternative to traditional reference air quality monitors. These sensors have the potential to provide high resolution air quality monitoring, both in time and space. Mallia et al. [16] highlighted how low-cost sensor networks can be used to characterize smoke plumes from wildfires and contribute to the evaluation of smoke transport models. Applications of low-cost sensor networks can also be extended to population exposure and health assessment [15,20].

The challenges that earlier research studies identified related to the use of low-cost particulate matter (PM) sensors were the lack of consistency between individual sensors, under or over reporting in comparison to reference methods and the deterioration in their performance over time [21,22]. Feinberg et al. [22] also called into question the consistency of the sensor performances based on meteorological conditions. The effects of elevated relative humidity (RH) and temperature on sensor performance is divided, with some studies showing negative effects of RH on sensor performance [23] while others show minimal impact [24,25,26]. Zamora et al. [27] highlighted that low-cost PM sensors are more accurate in environments with polydispersed particle sources and for PM with less than 1 μm diameter. Since then, more studies have investigated the performance of low-cost particle sensors under various particle loading scenarios and have established various calibration curves with or without environmental terms [26,28,29,30]. The Plantower PM sensors (PMS1003/3003/5003) have been tested against reference methods in several studies under laboratory conditions to assess the sensor’s suitability and accuracy [27,31,32,33,34,35,36,37,38] as well as in field-based measurements [20,24,25,26,29,30,39,40,41,42,43]. With the growing use of low-cost sensors both by the public and for research purposes, it is important to be able to interpret the data and understand their potential limitations [44].

With this understanding, we have conducted a number of field monitoring campaigns across regional Victoria. We selected locations impacted by both residential woodsmoke and planned burn emissions to evaluate the utility of the Plantower PMS3003 [45,46] for use in the detection and monitoring of biomass smoke as part of a broader Science, Technology, Engineering and Mathematics (STEM) project.

We present results from a range of validation monitoring tests that have been conducted using the sensor to demonstrate its utility for conducting biomass monitoring and to provide a better understanding of what can be interpreted from the data.

Objectives:

- Develop a smoke-specific calibration curve for the low-cost sensor SMOG units developed in this study and test how the calibrated data set for PM_2.5_ compares against gravimetric mass measurements and reference instruments at three different locations and during different seasons.
- Assess the suitability of low-cost particle sensors to detect ambient smoke events and provide insights into the spatial and temporal patterns of these events.
- Develop a school STEM curriculum focusing on the construction, deployment and analysis of data from low-cost particle sensors to assess biomass burning impacts on regional air quality where regulatory air quality monitoring is sparse.

## 2. Materials and Methods

### 2.1. Instruments

The SMoke Observation Gadget (SMOG) is a unit that comprises of a Plantower Laser PM_2.5_ dust sensor (model PMS3003) [45,46] and a digital temperature and humidity sensor (DHT22 AM2302). The PMS3003 uses a light scattering principle to measure particles suspended in the air. A red-coloured laser (wavelength of 650 ± 10 nm [45]) shines light into a measuring cavity through which air is drawn by a fan. A photo-diode measures scattered light at a 90 degrees angle to the laser beam. Flow is not critical because there is no size selective inlet or means of collecting aerosol for gravimetric calibration. The amount of scattered light is measured by a photodiode detector to determine the mass concentrations of particles using a proprietary algorithm based on Mie Theory. The measuring cavity is designed to be a light trap, so that only scattered light falls onto the receptor. The Plantower PMS3003 provides continuous measurements of PM_1_, PM_2.5_ and PM_10_ concentrations with a response time of 10 s. In this study, the raw standard PM_2.5_ output data from the PMS3003 (e.g., CF = 1) were used.

**Figure 1:**
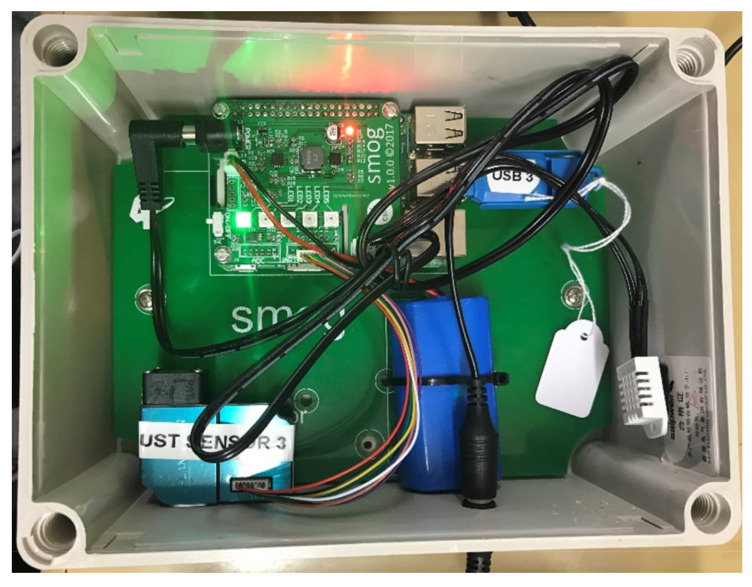
Assembled Smoke Observation Gadget (SMOG).

The SMOG unit has both a particle and temperature/humidity sensor that are interfaced via a printed circuit board (PCB) to a microcontroller, a Raspberry Pi Model 3B (RPi). The RPi receives information from the sensors and logs the data at 5-min intervals to an internal micro-SD card and an external USB stick. The electronics are consolidated onto a single PCB that connects directly to the RPi. All components are housed in a waterproof enclosure (150 × 200 × 100 mm) to facilitate outdoor deployment. Figure 1 shows an assembled SMOG unit. The unit also has a number of light-emitting diodes (LEDs). These indicate the operating status of the system at the start-up, and the particle load in the air during sampling. The overall design was simplified to ensure that Grade 6 students could construct and operate the monitor.

The SMOG units were tested at various locations and in different seasons alongside a tapered element oscillating microbalance (TEOM, Thermo Fisher Scientific, Waltham, MA, USA), an E-Sampler (Met One Instruments, Grants Pass, OR, USA) and a Fidas^®^ 200 S (Palas GmbH, Karlsruhe, Germany).

The TEOM is an EPA-designated equivalent method used for real-time measurements of the mass concentrations of particles. Air is drawn through a filter resting on a microbalance at a known flow rate and the change in frequency of an oscillating microbalance relative to the blank filter weight determines the volumetric concentration of particulate matter in ambient air [47]. The measurement is only dependent on the mass of the particles (i.e., not density, chemical composition or optical or electrical properties). A filter dynamic measurement system (FDMS) is used to adjust for the volatile component of the mass.

The E-sampler contains a diode laser that operates at a 670 nm wavelength and measures real-time particles through near-forward light-scattering. Accurate flow in the E-sampler is critical to ensure accurate size selection through the inlet for gravimetric calibration of PM_2.5_. The E-samplers operate at a flow rate of 2 L/min and collect both continuous particle measurements at a 5 min interval by light-scattering and particle mass on pre-weighed 47 mm Fluoropore membrane filters with a 1 μm pore size (Merck Millipore, Darmstadt, Germany). The scattering is converted into mass concentration using a gravimetric K-factor determined from the aerosol mass collected on a filter during sampling. Although the E-sampler is not an EPA-designated equivalent method, applying a gravimetric K-factor generated for the E-sampler during the measurement period ensures that accurate concentration measurements and good agreement with federal reference methods (FRM) and federal equivalent methods (FEM) are achieved.

The Fidas^®^ 200 S, a European equivalent reference method, is an optical aerosol spectrometer that continuously analyzes ambient particles present in the size range 180 nm–18 µm. It is equipped with a polychromatic LED light source and has a 90° scattering angle.

The TEOM, E-sampler and Fidas are all equipped with an aerosol drying system controlled by dynamic heating of the inlet to keep relative humidity of the intake air below 50%. In addition to removing water, heating the intake air has the undesired effect of evaporating volatile PM. The TEOM is the only instrument to control for this using a filter dynamics measurement system. Operational settings of the various types of particle instruments are provided in Table 1. It should be noted that differences in wavelengths of the light source and scattering angles between instruments may impact on the sensor response. The E-sampler and PMS3003 use a similar light source but a different scattering angle while the Fidas has a similar scattering angle to the PMS3003 but uses a different light source.

### 2.2. Measurement Locations

We used the data set collected during a peat fire event near Port Macquarie, NSW (latitude −31.4337, longitude 152.9153) to develop a smoke-specific calibration curve for the SMOG units. This event was chosen as it was a prolonged biomass burning particle pollution event that presented a wide range of particle concentrations. Two SMOG units were collocated alongside a standard regulatory method (FDMS-TEOM) between August and December 2019 to evaluate smoke impacts due to the nearby peat fires [48]. The monitoring equipment was set up at a mobile monitoring site operated by the NSW Department of Planning, Industry and Environment (DPIE). The site was located in the car park of a local library. Readings from the SMOG units were averaged on an hourly and 24 h basis for comparison with the TEOM and for the development of a smoke-specific calibration curve for the SMOG units.

A number of field-based measurements were conducted to test the suitability of the SMOG units to accurately detect and monitor biomass burning events in different locations and under different meteorological conditions (Table S1 and Figure S1).

Measurements were conducted on the rooftop of CSIRO laboratories at Aspendale, Victoria (latitude −38.025, longitude 145.102) located 30 km south of Melbourne and in close proximity (~50 m) to the Port Philip bay shoreline from 23 to 26 April 2018 (autumn) and between 25 June and 16 July 2018 (winter) (Figure S2). The area is impacted by local residential woodsmoke emissions during autumn/winter. In autumn, three SMOG units were compared to two collocated E-samplers fitted with a PM_2.5_ size-selective inlet and to the Fidas. In winter, one SMOG unit was collocated with an E-sampler fitted with a PM_2.5_ size-selective inlet and with the Fidas.

Ambient PM_2.5_ measurements using the low-cost sensor SMOG units were completed at fifteen locations in north-east Victoria between 1 May 2018 and 6 June 2018 and at six locations in north-east Victoria between November 2018 and June 2019 (Figure S1). The monitoring sites were located in areas that had the potential to be impacted by either prescribed burns, stubble burns or bushfires. Two SMOG units were deployed at each location. During each deployment period in north-east Victoria, reference instruments were installed at one location (e.g., Rutherglen in May/June 2018 and Alexandra between November 2018 and June 2019) to test the performance of the SMOG units either against the Fidas and/or the E-sampler.

The smoke-specific calibration curve parameters were applied to all deployed SMOG units with the hourly calibrated data set being compared against the reference instruments.

### 2.3. Data Analysis

A data cleaning criterion was applied to the raw data output from the SMOG units to remove erroneous data due to errors in PM filter sizing (e.g., check if PM_10_ ≥ PM_2.5_ ≥ PM_1_) and unrealistic spikes or drops in temperature and PM_2.5_. The filtered data set was then aggregated to hourly averaged data using a 75% data capture.

The limit of detection (LOD) for the SMOG units was determined by using the method of Wallace et al. [49]. LOD has been defined as the lowest concentration at which the ratio of the mean to standard deviation exceeds 3.

The following parameters were used to assess the performance of the SMOG units. Precision refers to how well all sensors reproduce the measurement of PM_2.5_ under identical circumstances. The precision of the SMOG units was evaluated using Pearson’s Correlation Coefficient to understand the associations between SMOG units. We used the reduced major axis linear regression relationships in R to provide insight into the pattern and extent of agreement between SMOG units [50]. A perfect agreement between SMOG units would show a slope of 1, indicating a similar response between the two instruments, and intercept of 0, indicating no bias in the sensor’s response. The intra-precision and accuracy of the SMOG units were also evaluated using Lin’s Concordance Correlation Coefficient (CCC). The CCC measures the agreement of continuous measurements obtained by two different methods by determining how far the observed data deviate from the line of perfect concordance (e.g., 1:1 line) [51,52]. The CCC value increases as a function of the accuracy of the data and the precision of the data. All statistical analysis was conducted using the statistical packages epiR in R [53].

The performance of the SMOG units was evaluated by computing linear regression and correlation with a reference instrument (e.g., E-sampler, Fidas or TEOM) using Ordinary Least Squares (OLS) Linear Regression in R [54]. This provided the strength of the relationship and the suitability of the calibration curve for the SMOG units. Lin’s concordance coefficient was also used to assess the precision and accuracy of the SMOG units relative to the reference instruments. Bland-Altman plots were used to examine the agreement between PM_2.5_ measurements made by the SMOG units and the corresponding reference particle instrument (R package ‘BlandAltmanLeh’ version 0.3.1) [55]. Bland-Altman diagrams (or difference plots) are typically used for the visual comparison of two measurements methods.

Additionally, the relative bias, mean absolute error (MAE), root mean square error (RMSE) and normalised root mean square error (NRMSE) were calculated for each hourly data set to measure data accuracy using R packages (Table S2).

### 2.4. Development of STEM Project

The SMOG units were designed to support a student-based project. The STEM project combined science, mathematics, engineering, and digital technology to address the issue of the impact of ambient particles from biomass burning sources on local and regional air quality and to assess air quality sensors. The project aimed to forecast smoke movements in smoke impacted regions and engage the community through schools.

The project was targeted at Grade 6–8 students and comprised of five lessons. The first three lessons were classroom based interactive presentations that taught students about air pollution and ambient particles, biomass burning and measurement techniques of ambient particles.

Lesson 4 was a hands-on session when students worked in teams to build the SMOG. After building the SMOG unit the students undertook a monitoring campaign using the SMOG units. Ideally, the monitoring campaign captured a period of potential high particle concentrations in the air such as those that result from prescribed burning in autumn or from domestic wood smoke in winter. The students were encouraged to set up the SMOG units outside their home and monitor the ambient air over approximately a 4-to-6-week period. Once the monitoring campaign was finished, the students returned their units to school to process and analyse their collected data set as part of lesson 5.

## 3. Results and Discussion

To evaluate the suitability of the SMOG units to capture and quantify smoke events, we assessed their performance against each other and against gravimetric mass measurements and reference instruments.

### 3.1. Development of Calibration Curve for SMOG Units

Previous studies have shown that the Plantower sensors correlated well with reference instruments, but they exhibited high biases [24,26,30,42,43,45]. In these studies, various simple and multi-variate linear regression curves as well as polynomial, exponential and quadratic correction equations were established for correcting PM_2.5_ concentrations.

**Figure 2:**
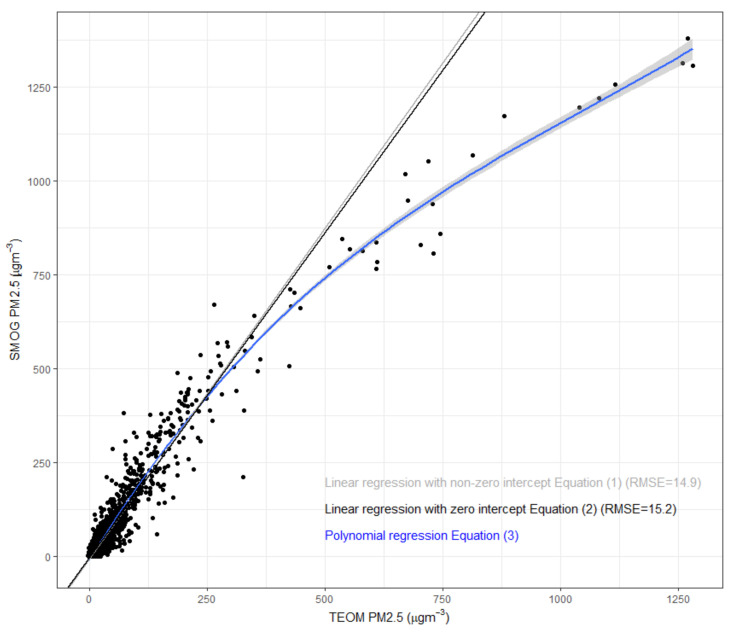
Fitted calibration curves for the SMOG units against the TEOM.

We used hourly PM_2.5_ measurements from a FDMS-TEOM during a period of smoke from peat fires near Port Macquarie (NSW) to develop a calibration curve for the SMOG units. Ambient hourly PM_2.5_ concentrations up to 1300 μg m^−3^ were measured during the sampling period between August–December 2019. Figure 2 shows the fitted lines for the two measurements methods, with the relevant equations shown below.

PM_2.5_ (μg m^−3^) = 1.667 + 0.569 × SMOG (1)

PM_2.5_ (μg m^−3^) = 0.578 × SMOG (2)

PM_2.5_ (μg m^−3^) = 5.45 + 0.45 × SMOG + 1.2 × 10^−4^ × SMOG^2^ + 1.8 × 10^−7^ × SMOG^3^ (3)

The data suggests that a linear relationship can be applied to correct the SMOG data up to an hourly PM_2.5_ concentration of ~300 μg m^−3^, with a 3rd degree polynomial curve best fitted for PM_2.5_ concentrations exceeding 300 μg m^−3^. Previous studies have shown non-linear behaviour above concentrations as low as 25 μg m^−3^ [43] and 40 μg m^−3^ [42], while other studies have shown a linear relationship exists at concentrations up to 125 μg m^−3^ [46], 150 μg m^−3^ [29] and 200 μg m^−3^ [26]. These differences could be due to particle composition, particle size and environmental conditions during testing, variations in the response of individual sensors or due to sensor algorithm [45,46,56].

Our linear equations with zero and non-zero intercepts showed similar slopes (1.73 and 1.76) and no significant difference in RMSE. This was also observed by Delp and Singer [29] who used simple scalars with no offset as adjustment factors for wildfire smoke. The median adjustment factors calculated for the Purple Air (PA) units that include Plantower sensors PMS5003 ranged from 0.42 to 0.58, in agreement with the adjustment factor of 0.58 in this study (defined as the slope in Equation (2)). The slopes we generated are also similar to the reported adjustment factor of 0.55 by Robinson [30] and the slopes by Holder et al. [26] (e.g., 0.51 for PA sensor and 0.57 for RAMP sensor). The slight difference in response between the PA and RAMP sensors (both units using the same Plantower PM sensor) was attributed to a potential difference in the sensor package design and/or post-processing algorithm [26].

A linear multivariate regression with additive terms for temperature and relative humidity was also developed for the SMOG PM_2.5_ concentrations in the 0–600 μg m^−3^ range.

PM_2.5_ (μg m^−3^) = 11.76 + 0.569 × SMOG − 0.056 × temperature (°C) − 0.157 × RH (%) (4)

PM_2.5_ (μg m^−3^) = 9.56 + 0.569 × SMOG − 0.142 × RH (%) (5)

A recent paper by Barkjohn et al. [28] developed an adjustment factor for the Purple Air Sensor that could be applied across the United States. After trialing a variety of model fits, the authors chose an additive RH model to work across a wide variety of data. Their fitted equation is similar to the one developed in this study (Equation (4)) with an approximate 8% difference. Holder et al. [26] observed little improvements in sensor accuracy when including an RH term and as a result recommended a simple regression without environmental terms.

### 3.2. PM_2.5_ Measurement Statistics

The LOD for PM_2.5_ measurements was calculated at 5.5 μg m^−3^, in agreement with the LOD of 6 µg m^−3^ reported by Barkjohn et al. [57] and Sayahi et al. [42] but higher than the LOD reported for PMS5003 [31,34,58].

There are several theories regarding the replacement of values below the LOD, either being censored or substituted with a constant value, such as half the LOD, the LOD divided by the square root of 2, or zero. Using either approach can have impacts on the analyses [59,60]. Depending on the percentage of values below the LOD, replacing values below the LOD can produce strong biases, questionable descriptive statistics and differences in correlation and regression relationships. Due to the variable number of values below the LOD, we retained the actual values to minimise introducing any bias in the analysis.

Tables S3–S5 show the summary statistics of the hourly measurements conducted in the field-based measurements. Boxplots of hourly PM_2.5_ concentrations for each measurement period and collocation of instruments are shown in Figure S3.

During the rooftop test at Aspendale, 35–43% of data were below the LOD, while for the field-based measurements in Northeast Victoria the percentage of hourly PM_2.5_ concentrations below LOD ranged from 32–89%. More than 90% of hourly PM_2.5_ concentrations remained below 25 μg m^−3^. Maximum hourly PM_2.5_ concentrations ranged from 32 to 220 μg m^−3^. As the maximum hourly PM_2.5_ concentration was measured at 220 μg m^−3^, we applied the linear calibration curve fitted through the origin to correct the hourly SMOG data for the field-based testing.

### 3.3. Inter-Comparison of SMOG Units

Intercomparison of collocated SMOG units enabled us to assess whether the SMOG units are suitable for measuring the spatial variability in PM_2.5_ concentrations. The summary of statistical metrics for the SMOG inter-comparisons is shown in Table 2.

Agreement between SMOG units 2 and 4 during the autumn measurements at Aspendale was very high (r^2^ = 0.98, slope = 1.03, intercept = 0.41) and CCC value of 0.98 (Table 2). The good agreement between the two SMOG units is shown in the Bland-Altman plot in Figure S4 (mean bias of 0.67 μg m^−3^ and agreement limits between −1.57 and 2.91 μg m^−3^). SMOG unit 3 did not perform as well with an under-reporting of ~20%, this was potentially due to drift in the sensor performance.

The collocation of two SMOG units at each of the sites in Northeast Victoria enabled us to further test the measurement agreement between SMOG units. The results were variable with very good agreement for most sites and units and very high CCC values indicating significant reliability (Table 2). We excluded any units for which sampling issues were identified, including unit 5 that showed much lower levels than other tested units, unit 20 that showed unrealistically high PM_2.5_ values likely due to an insect in the dust sensor which affected the sensor readings and unit 27 that switched off after 7 h. Poorer performance was observed at concentrations below 10 μg m^−3^. Examples of scatter and Bland-Altman plots are shown in Figure S5 highlighting the variable agreements. Possible explanations for the poorer performance between SMOG units include drift in sensor performance (as reported by Sayahi et al. [42] and Wang et al. [36]); misalignment of the sensor inlet with the inlet hole of the enclosure; very localized sources (e.g., SMOG2&4 in NE Victoria were deployed indoors and were potentially more sensitive to location).

The rooftop tests conducted at CSIRO Aspendale and the collocated SMOG units at north-east Victoria have shown an overall good intra-precision between SMOG units. Previous studies have shown very high correlations between PMS3003 units. For example, Zheng et al. [46] reported r^2^ values of 0.98–1.0 among five PMS3003 units, while Kelly et al. [45] showed high correlations (r^2^ > 0.99) between two PMS3003 units.

The data suggest that the SMOG units are reliable over a range of concentrations, temperatures (5–50 °C), and RH values (11–91%) which suggests they can be deployed to assess the spatial variability of PM_2.5_ concentrations in a wide range of locations and conditions.

### 3.4. Evaluation of PM_2.5_ Measurements Made by Optical Instruments versus Gravimetric Mass Measurements

Regression analysis of the continuous measurements by light-scattering versus gravimetric mass measurements for the E-sampler and Fidas are provided in Table 3 and Figure S6.

Comparison of gravimetric mass measurements against averaged corrected SMOG data showed ratios varying between 0.69 and 1.48 (excluding time periods with less than 50% data capture and more than 90% of data below LOD). The corrected SMOG data measured at Rutherglen were within 10% of the gravimetric mass measurements. Most of the SMOG data measured during the summer at Alexandra were below the LOD, with averaged corrected SMOG data being lower than the gravimetric mass measurements. In April, corrected SMOG data was in good agreement with the gravimetric mass measurements, while higher PM_2.5_ concentrations compared to gravimetric mass measurements were observed in winter (May–June) at Alexandra. A similar trend was observed for the measurements with the Fidas and E-sampler. Lower ratios were observed in winter at both Aspendale and Alexandra when high concentrations of particle events were attributed to residential wood smoke. This trend is in agreement with the findings by Kelly et al. [45] and Tryner et al. [35] who reported an overestimation in sensor data compared to TEOM data when the sensor was exposed to wood smoke.

The results in Table 3 show that the Fidas over-reported PM_2.5_ concentrations compared to gravimetric mass measurements, while the E-sampler under-reported PM_2.5_ concentrations compared to gravimetric mass measurements except for measurements completed in winter at Aspendale and Alexandra.

Systematic discrepancies between light-scattering monitors and reference methods have been observed in previous research studies [61,62,63]. The over-estimation in PM_2.5_ concentrations has been attributed to the differences between the optical properties of the manufacturer’s factory calibration particles and wood smoke particles and can be adjusted using a site-specific or season-specific calibration factor. The data also shows that there is not a uniform response of the light-scattering instruments to the different particle sources. This will be further explored in Section 3.6.

### 3.5. Performance Assessment of SMOG Units

**Figure 3:**
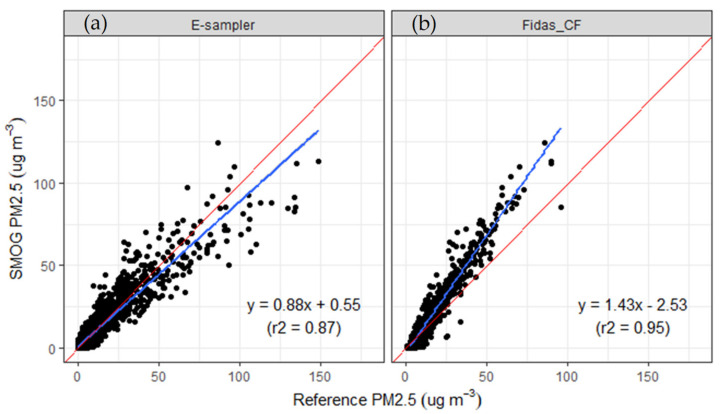
Scatterplot of hourly PM_2.5_ concentrations measured with the SMOG units compared to (**a**) the E-sampler and (**b**) Fidas_CF (both of which have been corrected against gravimetric mass measurements). Blue lines show linear least-squares fit; red line represents 1:1 line.

To check the accuracy of the SMOG units, we compared the hourly PM_2.5_ concentrations measured with the calibrated SMOG units using gravimetrically corrected measurements from the collocated Fidas (Fidas_CF) and E-sampler. Table 4 shows a summary of the statistical parameters using the different calibration curves for the SMOG calibration as defined in Section 3.1. The data shows that there is a minimal difference between the different calibration curves. Therefore, we used the calibration curve with zero intercept (Equation (2)) for the remaining analysis of the data sets. Table 4 also shows the statistical analysis of site- and season-specific intercomparison using Equation (2). Intercomparisons between calibrated SMOG (using Equation (2)), Fidas and E-sampler are shown in Figure 3.

**Figure 4:**
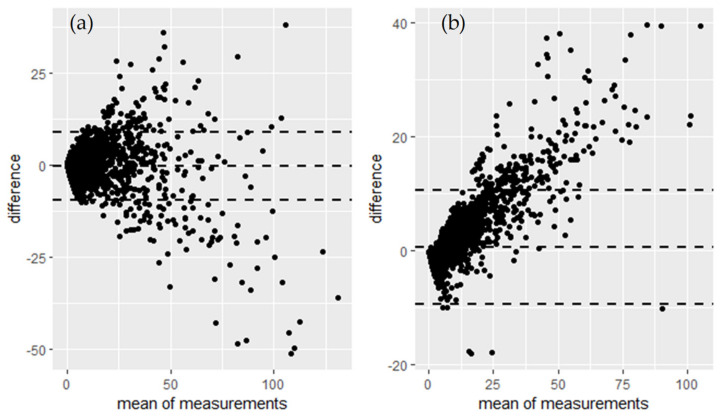
Bland-Altman plots of hourly PM_2.5_ concentrations measured with the SMOG units compared to (**a**) the E-sampler and (**b**) Fidas, both corrected against the gravimetric mass measurements.

The SMOG units showed very good correlation with the E-sampler, with a slope of 0.88, intercept of 0.55 and a CCC value of 0.93. The SMOG units exhibited a bias within 10 μg m^−3^ with a larger bias observed at higher ambient PM_2.5_ concentrations (Figure 4). This variability seems in agreement with the reported consistency of ±10 μg m^−3^ at concentrations ranging from 0–100 μg m^−3^ reported for the Plantower sensors [64]. At higher PM_2.5_ concentrations, the SMOG units reported lower PM_2.5_ concentrations compared to the E-sampler.

The correlation between the SMOG and Fidas was very high (r^2^ = 0.95), with an over-reporting compared to gravimetrically corrected Fidas data (slope of 1.43). Similarly to the E-sampler, the Bland-Altman plot (Figure 4) shows agreement limits within 10 μg m^−3^ and a proportional difference variability between the PM_2.5_ concentrations measured by the SMOG unit and the Fidas, with a widening trend of agreement as PM_2.5_ concentrations increased over 25 μg m^−3^. An increase in the absolute bias with increased ambient PM_2.5_ concentrations has also been reported by Gupta et al. [15].

Unlike the SMOG units, the E-sampler and Fidas are fitted with a drier in the inlet to remove excess water from the air and keep the relative humidity below 50%. As the SMOG sensors are based upon light scattering principles, particle hygroscopic properties can affect mass concentration estimations. Multivariate regression analysis conducted in this study has shown that changes in RH were significantly associated to changes in PM_2.5_. The association between temperature and PM_2.5_ concentrations was less consistent. However, using the multivariate regression analysis developed for the calibration curve of the SMOG vs. TEOM did not significantly alter the regression parameters and hence the sensor accuracy (Table 4). A similar observation was made by Holder et al. [26] who recommended using a simple linear regression to correct the data from the PA sensors, and other field-based evaluations of Plantower sensors [24,25,42]. The low insensitivity of the SMOG units to RH may be due to the low hygroscopicity of smoke particles [65].

**Figure 5:**
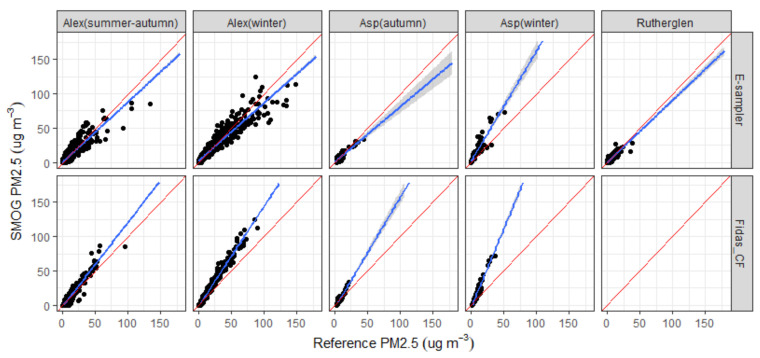
Scatterplot of hourly PM_2.5_ concentrations measured with the SMOG units compared to the E-sampler and Fidas_CF (both of which have been corrected against gravimetric mass measurements) by season and location. Blue lines show linear least-squares fit; red line represents 1:1 line.

Plotting the SMOG data vs. the reference data by location and season shows a closer fit for autumn at all locations compared to winter (Figure 5). Statistical metrics also showed a poorer agreement between SMOGs and reference instruments with a NRMSE above 100% observed during winter at Aspendale as well as higher RMSE and MAE values observed during winter at both Aspendale and Alexandra (Table 4).

Correlations between SMOG units and E-sampler during the Aspendale autumn campaign were high (r^2^ = 0.77–0.91), with an average slope of 0.80 and intercept of 0.68. The agreement between SMOG units and E-sampler was good (CCC values of 0.83 to 0.87). Performance of the SMOG unit against the Fidas was good, with a poorer performance for SMOG3 (possibly as a result of drift in sensor performance). Overall, the SMOG units over-reported by ~40% when using Fidas data corrected against gravimetric mass measurements.

The set of measurements conducted at Aspendale in winter 2018 showed high correlations between the SMOG unit and the E-sampler (r^2^ = 0.84, slope of 1.60 and an intercept of 1.67) and the Fidas (r^2^ = 0.96, slope of 2.29, intercept of −3.62). However, the SMOG units over-reported PM_2.5_ concentrations, with agreement limits ranging from −15.6 to 15.0 μg m^−3^ for the E-sampler and from −9.9 to 19 μg m^−3^ for the Fidas.

Good correlation (average r^2^ value of 0.76) was observed between the two E-samplers and the two SMOG units at the location in Rutherglen, Victoria with slopes ranging between 0.88 and 0.96. The scatter plots between SMOG7 and E-samplers indicate a large scatter when PM_2.5_ concentrations were below 10 μg m^−3^ and an underestimation of PM_2.5_ when concentrations were above 15 μg m^−3^ (Figure 5). The trend is similar to what was observed during autumn at Aspendale.

At Alexandra, we observed a larger scatter in the E-sampler vs. SMOG data set with slightly higher PM_2.5_ concentrations measured by the SMOGs in autumn and lower PM_2.5_ concentrations measured in winter. Less scatter is observed in the Fidas vs. SMOG data set with the SMOG data aligning with the corrected Fidas data up to a PM_2.5_ concentration of ~25 μg m^−3^ and an over-reporting in PM_2.5_ concentrations above 25 μg m^−3^ with the over-reporting more pronounced in winter compared to summer-autumn.

Overall, the SMOG units exhibited a high correlation with reference instruments (r^2^ > 0.75). Bias and error values were within recommended performance metrics [66], except for winter.

Observed correlations in this study were higher compared to other field-based studies using the Plantower sensor [67]. Moderate correlations (r^2^ of 0.40) were reported by Zheng for hourly PM_2.5_ measurements made with the Plantower PMS3003 sensor and an E-BAM reference instrument, with improved correlations when averaging times were increased to 6 h and 12 h [46]. Field-testing of three Plantower PMS3003 units against a BAM-1020 (Met one Instruments) conducted by the South Coast Air Quality Management District (SCAQMD) provided r^2^ values of 0.58 for hourly PM_2.5_ measurements [68]. Liu also reported moderate to good correlations (0.44–0.91) with performance varying by location and particle sources [40]. Poorer performance was observed for marine aerosols and fresh vehicle emissions with a better response to mixed urban background emissions, aged traffic emissions and industrial emissions. Higher correlations (r^2^ of 0.83–0.92) between PMS units and FEMs were reported in studies where biomass burning was the dominant particle source [26,29,45] as was the case in this study.

**Figure 6:**
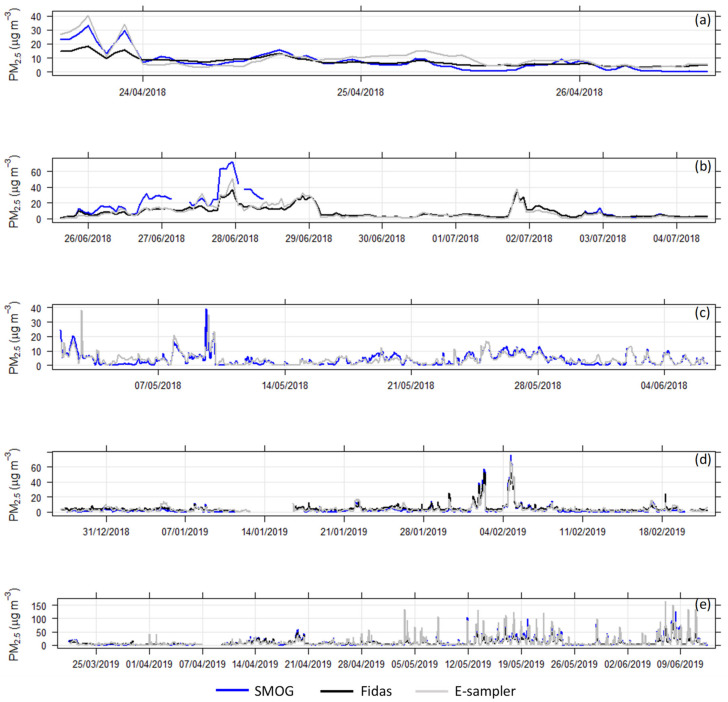
Hourly time series of ambient PM_2.5_ concentrations at the four measurement locations in different seasons: (**a**) Aspendale autumn, (**b**) Aspendale winter, (**c**) Rutherglen, (**d**) Alexandra summer, (**e**) Alexandra autumn-winter.

Time series plots of hourly PM_2.5_ concentrations measured with the calibrated SMOG units compared with a reference instrument (E-sampler/Fidas) corrected against gravimetric mass measurements are shown in Figure 6 for Aspendale autumn, Aspendale winter, Rutherglen and Alexandra summer and Alexandra autumn/winter.

The time series plots show that the SMOG units closely follow the PM_2.5_ trends of the collocated E-sampler and Fidas, indicating that the response time of the SMOG units is comparable to that of the E-sampler/Fidas and that peaks are temporally captured correctly.

### 3.6. Capturing Smoke Plume Events

At Aspendale in winter, we observed an elevated PM_2.5_ event on 27–28 June 2018 when the SMOG, Fidas and E-sampler were operational. Hourly PM_2.5_ concentrations remained above 25 μg m^−3^ for 15 h.

In Rutherglen, we observed a short spike in PM_2.5_ concentrations on 3 May 2018 and elevated PM_2.5_ concentrations between 8–10 May 2018. Elevated PM_2.5_ concentrations were measured at the other sampling sites during May 2018 and were either attributed to planned burns or stubble burns.

**Figure 7:**
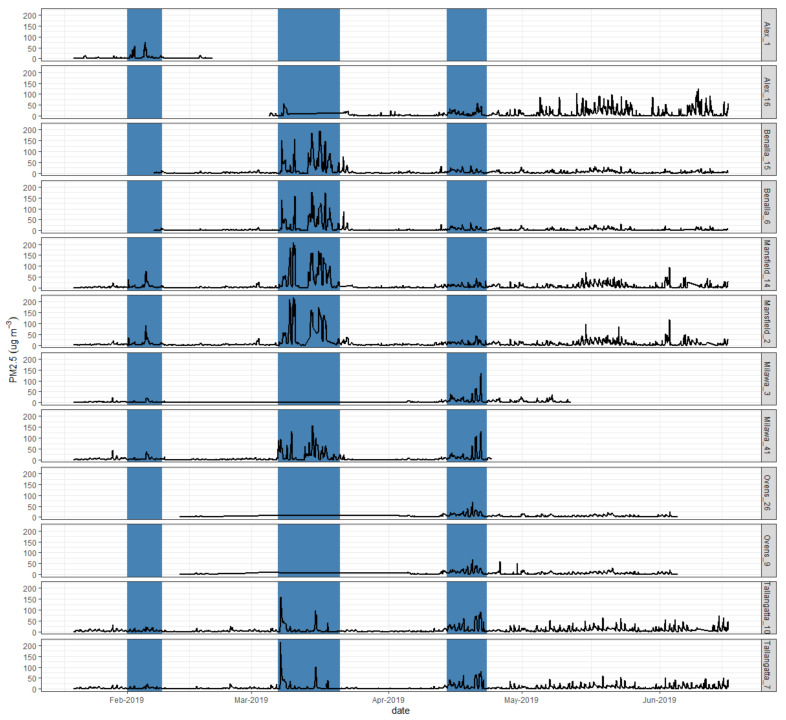
Time series plots of hourly PM_2.5_ concentrations measured at monitoring sites in NE Victoria in 2019. Shaded polygons represent the smoke plume events.

During the 2018/19 measurement period, three major PM_2.5_ peak events were identified: 1–8 February 2019, 7–20 March 2019 and 14–22 April 2019 (Figure 7). In early February, the Alexandra site recorded increased hourly PM_2.5_ concentrations with a maximum peak concentration of 75 µg m^−3^ recorded on 4 February. Increased PM_2.5_ concentrations were also measured at Mansfield, Milawa and Tallangatta. The elevated PM_2.5_ concentrations were likely due to fires to the southeast of Alexandra. On 13–18 March, smoke plumes from fires to the east impacted the monitoring sites at Mansfield, Benalla and Milawa. Tallangatta also showed increased PM_2.5_ concentrations during March but trends in PM_2.5_ concentrations differed to the other monitoring sites suggesting that smoke impacting Tallangatta originated from different fires. Elevated PM_2.5_ concentrations observed at monitoring sites in April are likely due to nearby planned burns. Maximum hourly PM_2.5_ concentrations that were measured during the 2018/19 field campaign ranged from 32 to 220 μg m^−3^.

Elevated PM_2.5_ concentrations were further observed during winter at Alexandra, Mansfield and Tallangatta and were most likely attributed to residential wood smoke.

Results on the performance of the SMOG units during the identified smoke plume events are provided in Table 5 and in Figures S7–S11. During smoke plume events from bushfires and planned burns, the corrected PM_2.5_ concentrations measured with the SMOG units were in good agreement with measurements with the Fidas or E-sampler (bias < 2 μg m^−3^, RMSE < 5 μg m^−3^). However, during winter, corrected SMOG PM_2.5_ concentrations were significantly higher compared to PM_2.5_ measurements made with the Fidas or E-sampler (bias of 5.9 to 13.3 μg m^−3^ and NRMSE of 94–132%).

Although Holder et al. [26] and Delp & Singer [29] reported variations in linear regression parameters between smoke impacted data sets, they applied a combined smoke adjustment factor which reduced the MAE and NRMSE for all data sets and resulted in minimal error (<20%). The data captured smoke plume events due to wildfires with likely similar particle properties.

Several research studies have shown that a combined calibration curve is suitable [26,28,29] while other studies argue for a seasonal or condition-specific calibration [20,42,69].

While we were able to investigate the effects of temperature and RH on the sensor performance other factors were not evaluated. One limitation of the study that could explain why the sensors responded differently between seasons is likely due to particle characteristics (e.g., composition and/or particle size). Mehadi et al. [33] showed an effect of EC fraction on PA sensor response, with a lower PA to reference ratio with increasing EC content. Kuula et al. [69] reported a stronger response of the PMS5003 sensor with an increased BC to PM ratio and also highlighted that accuracy of the PMS5003 sensor benefited from a residential wood smoke specific adjustment factor. As even small changes in EC/OC ratios can affect PM optical properties, the different chemical composition of residential wood smoke to smoke plumes from bushfires in summer and planned burns in autumn may be a contributing factor to the observed differences in the response of the optical instruments. The calibration curve developed in this study was determined from smoke plumes of peat fires which are likely to have a higher organic carbon content. The higher scattering efficiency of organic carbon compared to the high absorption efficiency of elemental carbon may explain the higher discrepancies between SMOG units and gravimetrically corrected reference instruments for the winter period at both Aspendale and Alexandra.

A larger scatter in the data was observed when comparing the SMOG data against the E-sampler data. This may be due to the different scattering angle between the E-sampler and Plantower sensor which affects the size range within which the oscillations are more pronounced [70] and/or the use of a cyclone for PM size separation vs. optical size separation. Zamora et al. [27] have shown that the sensors performed poorly when measuring particles in the size range of 2.5–5 μm while Kuula et al. [69] has shown better agreement for PM_1_ than for PM_2.5_ for wood smoke particles.

Like other educational programs using low-cost sensor technology [71], the SMOG units have proven to be a useful educational tool to teach students about particle sources and their impact on air quality. Approximately 85 Grade 5 and 6 students took part in the study with 41 SMOG units being built and deployed. The feedback from the pilot study was very positive among the students, teacher, and principal. Building the SMOG unit was the favourite activity. The students also liked that their collected data contributed to a larger scale project to further our understanding on biomass burning impacts in regional areas of Victoria.

## 4. Conclusions

We were able to conduct a number of field-based monitoring campaigns where the SMOG units were tested over a wide range of environmental conditions (e.g., temperature and RH range) and PM_2.5_ concentration ranges. This provided us with important data on the performance of the units under different meteorological conditions and in different locations with different biomass smoke sources.

Based on the sensor performance when testing a number of units simultaneously we are confident that the SMOG units can be used to increase spatial coverage of PM_2.5_ monitoring, as precision between SMOG units when regularly maintained was very high.

The field-based measurements suggest that the Plantower PMS3003 dust sensor can provide relevant information about ambient PM_2.5_ concentrations in an airshed impacted predominantly by biomass burning, provided that an adequate adjustment factor is applied. This study suggests that a uniform adjustment factor applied to sensor data may not be appropriate across all PM sources and that a residential wood smoke adjustment factor may need to be applied to increase the accuracy of the sensor.

The study also highlighted that sensor accuracy and precision may vary depending on reference instruments used for comparison purposes.

## Figures and Tables

**Table 1:** Operational settings of instruments.

Parameters	SMOG	E-Sampler	Fidas	TEOM
Sampling time (s)	1	1	1	2
Size range (μm)	0.3–10	0.1–100	0.18–18	NA
Resolution (μg m^−3^)	1	1	0.1	0.1
Effective detection range (μg m^−3^)	0–500	0–65,000	0–10,000	0–1,000,000
Flow rate (lpm)	NA	2	4.8	3
Temperature range (°C)	−10 to 60	−30 to 50	−20 to 50	−40 to 60
Humidity range (%)	0–99	Drying system	Drying system	Drying system
Light source wavelength	650 nm	670 nm	Polychromatic LED	NA
Scattering angle	90°	forward	85–95°	NA
PM sizing	Mie-scattering	Cyclone	Mie-scattering	PM_2.5_ inlet
Weight (kg)	0.04	6.4	9.3	18
Size (mm)	38 × 35 × 12	267 × 235 × 145	180.5 × 450 × 320	432 × 483 × 1400

**Table 2:** Statistical metrics of SMOG inter-comparison.

Measurement Period	SMOG ID	Slope	Intercept	r^2^	RMSE	NRMSE	Bias	MAE	CCC	N (h)
Aspendale autumn (2018)	2 vs. 3	0.84	0.95	0.94	2.06	26.4	0.33	1.71	0.95	72
	2 vs. 4	1.03	0.41	0.98	1.32	16.9	-0.67	0.96	0.98	72
	4 vs. 3	0.81	0.62	0.94	2.43	28.7	1.00	1.93	0.94	72
NE Victoria May–June 2018	23 vs. 25	0.98	−0.01	0.98	0.70	20.0	−0.09	0.39	0.99	831
	15 vs. 21	1.00	0.15	0.98	0.67	15.5	0.14	0.36	0.99	775
	3 vs. 24	1.03	−0.10	0.74	3.07	82.6	0.01	1.24	0.86	792
	1 vs. 30	0.99	−0.77	0.97	1.76	26.5	−0.84	1.03	0.98	614
	22 vs. 28	0.82	−0.10	0.93	1.57	51.1	−0.82	0.87	0.92	820
	6 vs. 7	0.99	−0.08	0.99	0.63	9.7	−0.15	0.39	1.00	72
	12 vs. 13	0.96	−0.05	0.99	0.74	16.8	−0.24	0.41	0.99	855
	2 vs. 4	0.85	−0.16	0.93	1.18	64.2	−0.51	0.52	0.94	855
	16 vs. 17	1.09	0.27	0.99	0.93	22.2	0.60	0.61	0.99	828
	18 vs. 19	0.97	0.04	0.99	0.85	11.9	−0.15	0.52	1.00	825
	8 vs. 9	0.97	0.74	0.97	1.98	22.4	0.53	0.80	0.98	849
	10 vs. 11	0.97	−0.34	0.99	1.33	18.5	−0.59	0.84	0.99	855
NE Victoria November 2018–June 2019	6 vs. 15	0.93	−0.25	0.99	2.12	25.7	−0.64	0.88	0.99	2502
	2 vs. 14	1.04	−0.19	0.99	1.91	76.0	0.13	0.77	1.00	2573
	3 vs. 41	0.91	−0.44	0.93	2.45	48.3	−0.80	1.25	1.00	1607
	9 vs. 26	1.11	−0.09	0.86	2.72	45.9	0.45	1.02	1.00	1384
	7 vs. 10	0.96	−0.41	0.96	2.22	25.7	−0.63	0.96	0.98	3360

**Table 3:** Comparison between gravimetric PM_2.5_ mass concentrations and PM_2.5_ concentrations measured using optical instruments, including raw and calibrated SMOG data.

Location	Date	Gravimetric	SMOG					Fidas			E-Sampler		
		(μg m^−3^)	Average ^1^ (μg m^−3^)	Average (OLS) ^2^ (μg m^−3^)	CF ^3^	Missing Data (%)	<LOD (%)	Average (μg m^−3^)	CF	Missing Data (%)	Average (μg m^−3^)	CF	Missing Data (%)
Aspendale	25/06/18–02/07/18	9.34	39.3	22.7	0.41	64	4	17.9	0.52	0	12.1	0.77	0
	02/07/18–09/07/18	3.42	na ^4^	na	na	na	na	7.4	0.46	0	4.8	0.71	0
	09/07/18–16/07/18	7.25	na	na	na	na	na	10.8	0.67	0	4.9	1.49	0
Rutherglen ^5^	01/05/18–21/05/18	4.71	7.12	4.12	1.15	3.8	69	na	na	na	3.59	1.31	0
	21/05/18–06/06/18	4.41	6.90	3.99	1.10	6.1	64	na	na	na	3.39	1.30	0
	01/05/18–21/05/18	4.60	7.12	4.12	1.12	3.8	69	na	na	na	4.00	1.15	0
	21/05/18–06/06/18	4.33	6.90	3.99	1.08	6.1	64	na	na	na	3.55	1.22	0
Alexandra	29/11/18–09/12/18	4.44	1.63	0.94	4.71	10	92	4.83	0.92	11	1.85	2.40	0
	18/12/18–27/12/18	3.77	6.79	3.92	0.96	81	68	4.20	0.90	4.7	2.02	1.87	0
	27/12/18–02/01/19	5.30	2.82	1.63	3.26	1.4	99	6.64	0.80	0	2.69	1.97	1.4
	02/01/19–12/01/19	4.48	2.75	1.59	2.82	11	95	5.85	0.77	13	2.71	1.65	0
	16/01/19–06/02/19	6.76	8.75	5.05	1.34	2.0	79	11.8	0.58	0	5.13	1.32	0
	06/02/19–04/03/19	4.52	2.88	1.66	2.72	49	91	6.84	0.66	2.5	2.82	1.60	2.7
	21/03/19–05/04/19	4.70	5.48	3.17	1.48	0	87	7.62	0.62	0.6	3.88	1.21	0.6
	05/04/19–18/04/19	7.32	13.2	7.62	0.96	26	54	15.6	0.47	33	5.86	1.25	20
	18/04/19–16/05/19	7.36	16.15	9.33	0.79	0	57	15.5	0.48	0	9.85	0.75	0
	16/05/19–13/06/19	12.03	30.2	17.4	0.69	3.1	46	24.4	0.49	0.1	18.1	0.66	0

^1^ Averaged non-calibrated PM_2.5_ concentration. ^2^ Averaged calibrated PM_2.5_. Concentration (using linear regression fitted through origin). ^3^ CF (calibration factor) = Gravimetric PM_2.5_ mass concentration/light scattering averaged PM_2.5_ concentration. ^4^ No data available from the SMOG units. ^5^ Fidas was not installed at the Rutherglen site.

**Table 4:** Performance of calibrated SMOG units in comparison to gravimetrically corrected reference instruments.

SMOG Calibration	Reference Instrument	Slope	Intercept	r^2^	Slope (Zero Int)	r^2^	RMSE	NRMSE (%)	MBE	MAE
Linear with zero intercept (all data) (Equation (2))	E-sampler Fidas	0.88 1.43	0.55 −2.53	0.87 0.95	0.90 1.30	0.90 0.94	4.76 5.17	67.3 74.6	−0.29 0.52	2.50 2.98
Linear with non-zero intercept (all data) (Equation (1))	E-sampler Fidas	0.87 1.40	2.15 −0.88	0.87 0.95	0.94 1.36	0.90 0.96	4.90 5.33	69.3 76.9	1.20 2.00	2.82 2.69
Polynomial (all data) (Equation (3))	E-sampler Fidas	0.71 1.15	5.71 3.23	0.88 0.95	0.89 1.32	0.83 0.95	6.39 5.22	90.3 75.3	3.66 4.31	5.03 4.40
Linear with additive RH term (all data) (Equation (5))	E-sampler Fidas	0.82 1.33	3.17 0.58	0.83 0.94	0.91 1.36	0.86 0.96	5.81 5.33	82.2 76.9	1.84 2.93	3.84 3.49
Linear with additive RH and temperature term (all data) (Equation (4))	E-sampler Fidas	0.82 1.34	3.40 0.72	0.84 0.94	0.93 1.37	0.86 0.96	5.77 5.42	81.6 78.2	2.11 3.12	3.88 3.56
Aspendale autumn (Equation (2))	E-sampler Fidas	0.80 1.61	0.68 −3.80	0.78 0.93	0.85 1.24	0.90 0.94	4.02 3.45	41.5 45.2	−1.22 0.83	3.42 2.33
Aspendale winter (Equation (2))	E-sampler Fidas	1.60 2.29	1.67 −3.62	0.84 0.96	1.69 2.04	0.91 0.97	11.0 11.9	139 146	6.63 6.80	6.84 7.25
Rutherglen (Equation (2))	E-sampler	0.90	0.12	0.76	0.92	0.87	2.08	50.0	−0.27	1.41
Alexandra summer-autumn (Equation (2))	E-sampler Fidas	0.88 1.23	0.05 −2.18	0.81 0.92	0.88 1.05	0.85 0.91	3.66 2.81	74.5 54.7	−0.56 −0.96	2.13 2.07
Alexandra winter (Equation (2))	E-sampler Fidas	0.84 1.45	2.54 −1.25	0.91 0.98	0.89 1.41	0.94 0.99	7.81 8.66	41.5 64.8	−0.26 4.53	4.22 5.50

**Table 5:** Summary statistics of hourly PM_2.5_ concentrations measured during smoke plume events.

Location	Date	Units	SMOG PM_2.5_ Range (μg m^−3^)	Slope	r^2^	Bias (Limit)	RMSE (μg m^−3^)	NRMSE
Aspendale	26–28 June 2018	SMOG vs. E-sampler_CF SMOG vs. Fidas_CF	5.0–72.4	1.69 ± 0.07 2.10 ± 0.04	0.91 0.98	11.5 (−8.1 to 31.1) 13.3 (−6.3 to 33.0)	15.2 16.6	105 132
Rutherglen	7–11 May 2018	SMOG vs. E-sampler4_CF SMOG vs. E-sampler5_CF	0.0–39.3	**0.82 ± 0.03** **0.83 ± 0.03**	0.88 0.87	−0.7 (−6.4 to 4.9) −0.5 (−6.3 to 5.3)	**2.96** **2.99**	50.5 53.2
Alexandra	1–6 February 2019	SMOG vs. E-sampler_CF SMOG vs. E-sampler_OLS SMOG vs. Fidas_CF SMOG vs. Fidas_OLS	0.1–75.3	**0.90 ± 0.02** **1.14 ± 0.02** **1.11 ± 0.02** **1.16 ± 0.02**	0.96 0.96 0.97 0.97	−1.2 (−10.5 to 8.1) 1.6 (−7.5 to 10.8) −0.25 (−8.3 to 7.8) 0.3 (−8.5 to 9.1)	**4.87** **4.93** **4.12** **4.48**	36.6 47.2 33.3 38.0
Alexandra	10–23 April 2019	SMOG vs. E-sampler_CF SMOG vs. Fidas_CF	0.0–57.3	**0.97 ± 0.03** **1.22 ± 0.02**	0.88 0.97	0.7 (−7.2 to 8.7) 1.3 (−4.2 to 6.8)	**4.10** **3.10**	46.8 37.8
Alexandra	12 May–13 June 2019	SMOG vs. E-sampler_CF SMOG vs. E-sampler_OLS SMOG vs. Fidas_CF SMOG vs. Fidas_OLS	0.0–125	1.36 ± 0.01 **0.89 ± 0.01** 1.59 ± 0.01 1.41 ± 0.01	0.93 0.94 0.99 0.99	5.89 (−10.5 to 22.3) −0.26 (−15.6 to 15.0) 5.9 (−11.5 to 23.4) 4.5 (−9.9 to 19.0)	10.2 7.81 10.7 8.66	89.4 44.4 94.1 67.6

Bold is used to easily identify where sensor performance was good.

## Data Availability

Not applicable.

## Supplementary Materials

The following are available online at https://www.mdpi.com/article/10.3390/s21217206/s1. Table S1. Deployment details of field-based measurements; Table S2. Data analysis parameters and equations; Table S3. Summary statistics of hourly PM_2.5_ concentrations measured during the rooftop tests at CSIRO Aspendale in 2018; Table S4 Summary statistics of hourly PM_2.5_ concentrations measured in Northeast Victoria in 2018; Table S5. Summary statistics of hourly PM_2.5_ concentrations measured in Northeast Victoria in 2018/2019; Figure S1. Location of the field-based measurement sites Figure S2. Set-up for intercomparison between SMOG units and E-samplers fitted with a PM_2.5_ size selective inlet; Figure S3. Boxplots for PM_2.5_ hourly concentrations at (a) Aspendale Autumn, (b) Aspendale winter, (c) Rutherglen (Victoria) and (d) Alexandra (Victoria). Whiskers denote the 10th and 90th percentiles; Figure S4. Bland-Altman plot of the agreement between PM_2.5_ concentrations measured by two SMOG units during autumn at Aspendale; Figure S5. Scatter plot and Bland-Altman plots of the agreement between PM_2.5_ concentrations measured by two SMOG units in Northeast Victoria; Figure S6. Linear regression analysis of optical measurements vs. gravimetric mass measurements; Figure S7. High particle event in winter at Aspendale; Figure S8. High particle event in autumn at Rutherglen; Figure S9. High particle event in summer at Alexandra; Figure S10. High particle event in autumn at Alexandra; Figure S11. High particle event in winter at Alexandra.
