# Supplementary material for: Performance and Deployment of Low-Cost Particle Sensor Units to Monitor Biomass Burning Events and Their Application in an Educational Initiative
Source: Sensors (Basel). 2021 Oct 29;21(21):7206. doi: 10.3390/s21217206 (PMC8588471; doi:10.3390/s21217206)
Supplement: Supplementary file 1 [file sensors-21-07206-s001.zip › sensors-1431934-supplementary.pdf]

Article

Performance and Deployment of Low-Cost Particle Sensor Units to Monitor Biomass Burning Events and Their Application in an Educational Initiative

Fabienne Reisen [1,*], Jacinta Cooper [1], Jennifer C. Powell [1], Chris Roulston [1] and Amanda J. Wheeler [2,3]

Table S1. Deployment details of field-based measurements.

Location	Sampling period	Season	SMOG units	High grade instrument	Temperature (°C) Average (range)	Relative Humidity (%) Average (range)
Aspendale, Victoria	23-26 Apr 2018	Autumn	3	E-sampler, Fidas	21 (15-29)	71 (19-92)
Aspendale, Victoria	25 Jun-16 Jul 2018	Winter	1	E-sampler, Fidas	14 (5-21)	59 (21-80)
NE Victoria	1 May-6 Jun 2018	Autumn/Winter	30	NA	NA	NA
Rutherglen, Victoria	1 May-6 Jun 2018	Autumn/Winter	2	E-sampler	13 (6-29)	59 (23-85)
NE Victoria	Nov 2018-Jun 2019	Summer/Autumn/Winter	12	NA	NA	NA
Alexandra, Victoria	Nov 2018-Feb 2019	Summer	1	E-sampler, Fidas	22.5 (5-43)	58 (9-98)
Alexandra, Victoria	Mar/Apr 2019	Autumn	1	E-sampler, Fidas	16.3 (2-38)	64 (13-98)
Alexandra, Victoria	May/Jun 2019	Winter	1	E-sampler, Fidas	10.0 (1-21)	86 (38-100)

Table S2. Data analysis parameters and equations

Statistical indicator	Definition
Root mean square error (RMSE)	$RMSE = \left(\frac{\sum(Ref_i - Sensor)^2}{n}\right)^{1/2}$
Normalised Root mean square error (NRMSE)	$NRMSE = \frac{RMSE}{\overline{Ref}}$
Relative bias	$Bias\ (\%) = \frac{Sensor - Ref}{Ref} \times 100\%$
Mean Absolute Error (MAE)	$MAE = \frac{1}{n}\sum \lvert Ref_i - Sensor_i \rvert$
Pearson correlation coefficient r	$r = \frac{N\left(\sum_{i=1}^{N} Sensor_i\, Ref_i\right) - \left(\sum_{i=1}^{N} Sensor_i\right)\left(\sum_{i=1}^{N} Ref_i\right)}{\sqrt{\left[N\left(\sum_{i=1}^{N} {Sensor_i}^2\right) - \left(\sum_{i=1}^{N} Sensor_i\right)^2\right]\left[N\left(\sum_{i=1}^{N} {Ref_i}^2\right) - \left(\sum_{i=1}^{N} Ref_i\right)^2\right]}}$
Lin's Concordance Correlation Coefficient (CCC)	$\rho_c = \frac{2\rho\sigma_x\sigma_y}{\sigma_x^2+\sigma_y^2+(\mu_x-\mu_y)^2}$, μ_x and μ_y are the means, σ_x^2 and σ_y^2 are the variances, ρ is the correlation coefficient

Table S3. Summary statistics of hourly $PM_{2.5}$ concentrations measured during the rooftop tests at CSIRO Aspendale in 2018 (QA/QC calibrated data set).
(Good AQ $PM_{2.5}$<25 μg m^{-3}, Fair AQ $PM_{2.5}$=25-50 μg m^{-3}, Poor AQ>50 μg m^{-3})

Date	Units	Mean	Median	Min	Max	RH (%)	%obs when RH>70%	Temperature (°C)	N	<LOD (%)	Good AQ (%)	Fair AQ (%)	Poor AQ (%)
23-26 Apr	SMOG2	7.8	5.9	0.05	32.8	55.0 (21-79)	33.3	22.1 (12-41)	73	43	95.8	4.2	0.0
	SMOG3	7.5	6.3	0.9	31.8	55.1 (21-78)	33.3	22.0 (13-40)	73	40	97.2	2.8	0.0
	SMOG4	8.5	6.8	0.2	34.0	53.7 (23-80)	31.9	22.0 (12-42)	73	35	94.4	5.6	0.0
	E-sampler4	9.7	7.5	2.3	44.9	42.7 (22-49)	NA	21.4 (15-28)	73	38	91.7	8.3	0.0
	E-sampler5	10.2	8.2	1.9	44.8	42.0 (21-49)	NA	21.6 (15-29)	73	36	91.7	8.3	0.0
	Fidas	13.8	11.1	4.2	39.3		NA		73	11	88.9	11.1	0.0
25Jun-4Jul	SMOG	14.9	8.0	0.01	72.4	57.9 (21-80)	32.3	13.7 (6-37)	105	40	77.5	16.7	5.9
	E-sampler_CF	8.2	4.8	0.00	51.3	45.3 (27-49)	NA	13.6 (5-21)	212	55	93.1	5.9	1.0
	Fidas_CF	8.1	5.4	0.8	37.2		NA		212	50	94.1	5.9	0.0

Table S4 Summary statistics of hourly $PM_{2.5}$ concentrations measured in Northeast Victoria in 2018.

(Good AQ $PM_{2.5}$<25 μg m^{-3}, Fair AQ $PM_{2.5}$=25-50 μg m^{-3}, Poor AQ>50 μg m^{-3})

Location	Date	Units	Mean	Median	Max	RH (%)	%obs when RH>70%	Temperature (°C)	N (hrs)	<LOD (%)	Good AQ (%)	Fair AQ (%)	Poor AQ (%)
Rutherglen region	1May-6June	SMOG23	6.3	4.8	66.0	49.3 (13-79)	5.9	16.7 (6-42)	837	56	99.4	0.6	0.0
		SMOG25	6.4	4.7	63.0	47.4 (15-74)	2.4	16.3 (6-39)	835	56	99.2	0.8	0.0
	1May-6June	SMOG5*	3.8	3.0	15.2	52.1 (24-68)		14.4 (6-30)	794				
		SMOG29	7.1	5.4	81.0	56.8 (25-75)	14.4	14.0 (6-29)	787	51	99.0	0.9	0.1
	1May-6June	SMOG15	7.1	5.9	30.7	57.3 (23-76)	14.3	13.5 (6-30)	794	46	99.9	0.1	0.0
		SMOG21	6.9	5.7	32.2	55.9 (22-74)	8.9	13.6 (6-31)	790	49	99.9	0.1	0.0
	1May-6June	SMOG26	6.3	4.6	39.9	49.1 (25-65)	0	14.9 (6-32)	799	60	99.2	0.8	0.0
	1 May	SMOG27	10.5	11.0	12.7	35.5 (31-41)		18.2 (15-25)	7				
	1May-6June	SMOG3	6.4	4.5	69.6	55.2 (23-73)	4.0	14.4 (6-29)	821	62	98.8	1.1	0.1
		SMOG24	6.4	4.4	44.2	56.6 (25-75)	9.8	14.2 (6-29)	810	62	98.5	1.5	0.0
	1May-6June	SMOG1	9.8	7.3	93.1	51.7 (7-84)	16.1	16.7 (6-57)	830	37	95.6	3.2	1.2
		SMOG30	10.7	8.2	98.0	46.0 (11-76)	2.6	16.7 (6-50)	835	34	96.4	2.6	1.0
	2May-6June	SMOG22	5.8	4.6	28.6	48.1 (22-66)	0	16.6 (7-32)	821	62	99.9	0.1	0.0
		SMOG28	6.6	5.2	29.8	45.0 (22-61)	0	16.8 (7-32)	821	53	99.9	0.1	0.0
DEDJTR	1-4May	SMOG6	6.5	5.7	27.6	39.0 (23-72)	5.6	19.2 (14-28)	72	47	98.6	1.4	0.0
	1May-6June	SMOG7	3.9	2.5	39.3	60.6 (24-85)	26.0	12.9 (6-29)	823	72	99.6	0.4	0.0
		E-sampler4	4.4	3.6	38.2		NA				99.7	0.3	0.0
		E-sampler5	4.4	3.6	38.5		NA				99.6	0.4	0.0
Winton Wetlands	1-3May	SMOG14#	30.8	24.3	131.1	29.7 (16-41)		21.7 (14-35)	34				
	1May-6June	SMOG20	6.6	4.7	39.2	47.9 (19-76)	3.4	14.8 (6-35)	836	58	98.3	1.7	0.0
Benalla	1May-6June	SMOG12	7.0	5.0	48.0	46.8 (22-63)	0	16.1 (10-28)	855	53	98.8	1.2	0.0
		SMOG13	7.3	5.1	73.7	45.2 (22-61)	0	16.4 (11-28)	856	53	98.9	1.1	0.0
Benalla (in-side)	1May-6June	SMOG2	4.7	3.3	41.7	32.2 (21-44)	0	21.9 (15-28)	867	76	99.9	0.1	0.0
		SMOG4	5.2	3.8	79.2	31.0 (23-40)	0	22.1 (15-28)	856	71	99.9	0.1	0.0
Ovens	1May-6June	SMOG16	6.8	5.2	48.1	40.6 (23-55)	0	19.7 (13-28)	829	56	97.3	2.7	0.0
		SMOG17	6.3	4.7	44.3	42.9 (24-57)	0	19.6 (12-28)	828	60	97.6	2.4	0.0

Bee-chworth	1May-6June	SMOG18	9.6	7.7	57.1	53.3 (21-72)	0.6	14.4 (6-35)	838	32	95.9	3.1	1.0
		SMOG19	9.7	7.9	56.0	50.3 (15-70)	0	15.2 (6-41)	833	32	95.6	3.4	1.1
Mansfield	1May-6June	SMOG8	11.1	8.1	79.5	49.1 (20-66)	0	15.0 (6-28)	867	37	91.8	7.3	0.9
		SMOG9	10.7	7.5	109.5	46.3 (20-62)	0	14.7 (6-28)	855	38	92.6	6.6	0.8
Tallangatta	1May-6June	SMOG10	9.6	6.6	60.9	46.3 (23-70)	0	16.6 (7-28)	855	44	94.3	4.9	0.8
		SMOG11	10.2	7.1	63.5	48.4 (22-73)	0.9	16.2 (7-28)	855	39	93.5	5.7	0.8

* Issue with SMOG unit (measured $PM_{2.5}$ concentrations much lower than collocated SMOG unit)

Issue with SMOG unit (likely insect in sensor chamber causing elevated PM readings

Table S5. Summary statistics of hourly $PM_{2.5}$ concentrations measured in Northeast Victoria in 2018/2019.

Good AQ $PM_{2.5}$<25 µg m^{-3}, Fair AQ $PM_{2.5}$=25-50 µg m^{-3}, Poor AQ>50 µg m^{-3})

Location	Date	Units	Mean	Median	Max	RH (%)	%obs when RH>70%	Temperature (°C)	N (hrs)	<LOD (%)	Good AQ (%)	Fair AQ (%)	Poor AQ (%)
Alexandra	1Dec18-20Feb19	SMOG1	2.8	1.2	75.3	36.9 (7-83)	2.1	31.1 (8-62)	1420	89	98.3	1.1	0.6
	5Mar19-16Jun19	SMOG16	10.7	3.4	125	57.5 (10-90)	25.5	17.1 (6-45)	2105	58	86.8	9.1	4.1
		E-sampler4	7.6	2.9	164		NA				92.6	4.6	2.8
		Fidas	12.4	6.6	173		NA				88.2	7.4	4.4
		Fidas_ols	6.8	3.6	95.4		NA				94.7	4.3	0.9
Benalla	7Feb19-16Jun19	SMOG6	5.6	1.8	175	43.4 (8-76)	1.5	19.7 (6-46)	3065	77	94.6	2.3	3.1
	7Feb19-16Jun19	SMOG15	7.1	2.1	196	45.3 (11-75)	1.3	20.1 (7-44)	3093	73	94.1	2.7	3.3
Mansfield	17Jan19-16Jun19	SMOG2	8.5	2.5	220	45.5 (5-85)	7.0	21.5 (6-52)	3456	69	91.5	5.0	3.5
	17Jan19-16Jun19	SMOG14	8.8	2.4	207	45.3 (7-94)	10.0	21.3 (6-51)	3491	70	91.4	4.8	3.8
Milawa	24Dec18-14Feb19/ 5Apr19-11May19	SMOG3	3.6	1.5	134	41.8 (3-100)	12.8	29.0 (6-58)	2123	83	98.2	1.1	0.7
	24Dec18-24Apr19	SMOG41	5.6	1.7	158	46.2 (7-100)	18.9	27.0 (6-54)	2880	80	94.6	3.0	2.4
Ovens	13Feb19-4Jun19	SMOG9	5.6	3.5	69.1	54.5 (2-100)	42.0	18.6 (6-48)	2342	63	95.2	3.5	1.3
	13Feb19-4Jun19	SMOG26	5.3	3.4	68.2	60.3 (4-100)	54.6	18.7 (6-48)	2316	64	96.1	3.0	0.9
Tallangatta	1Dec19-16Jun19	SMOG7	5.1	2.1	213	49.7 (10-99)	20.0	22.7 (6-50)	4292	75	95.5	3.2	1.3
	1Dec19-16Jun19	SMOG10	5.5	2.3	159	42.5 (2-81)	3.3	24.9 (6-64)	4633	73	95.3	3.4	1.4

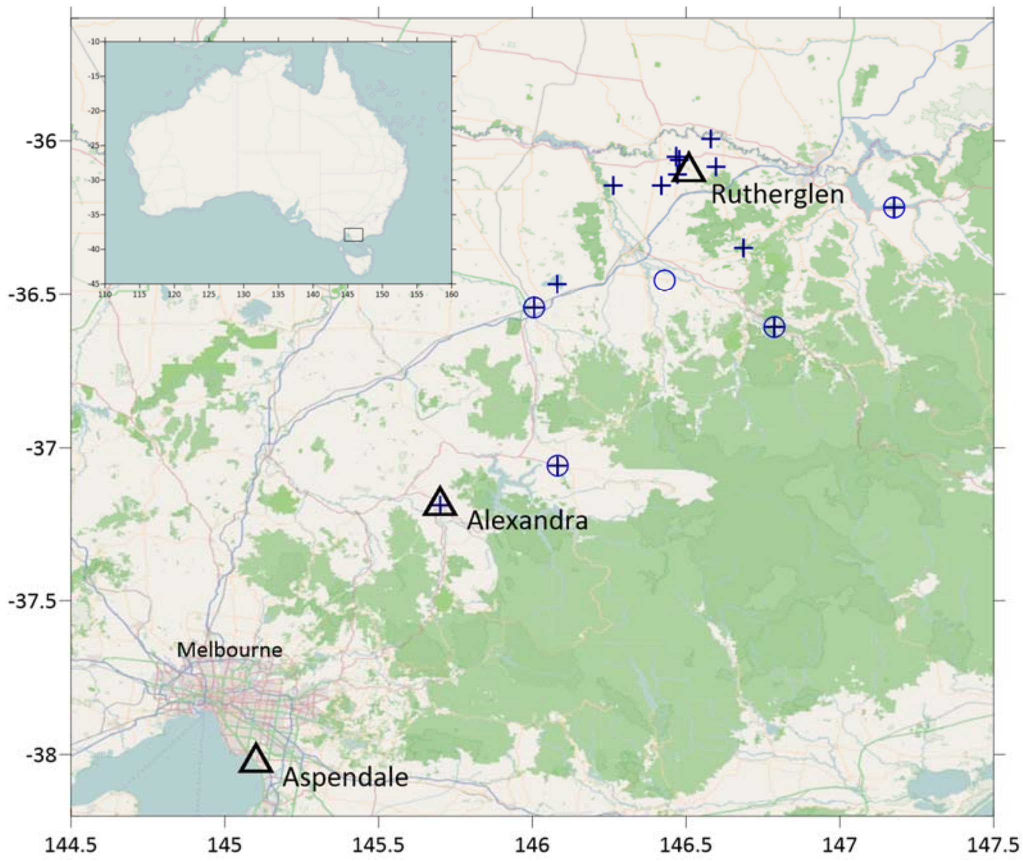

Figure S1. Location of the field-based measurement sites: Δ represent sites with collocated reference instruments; + represent North-east Victoria measurement sites (sampling period of May-June 2018), O represent North-east Victoria measurement sites (sampling period Nov 2018-June 2019)

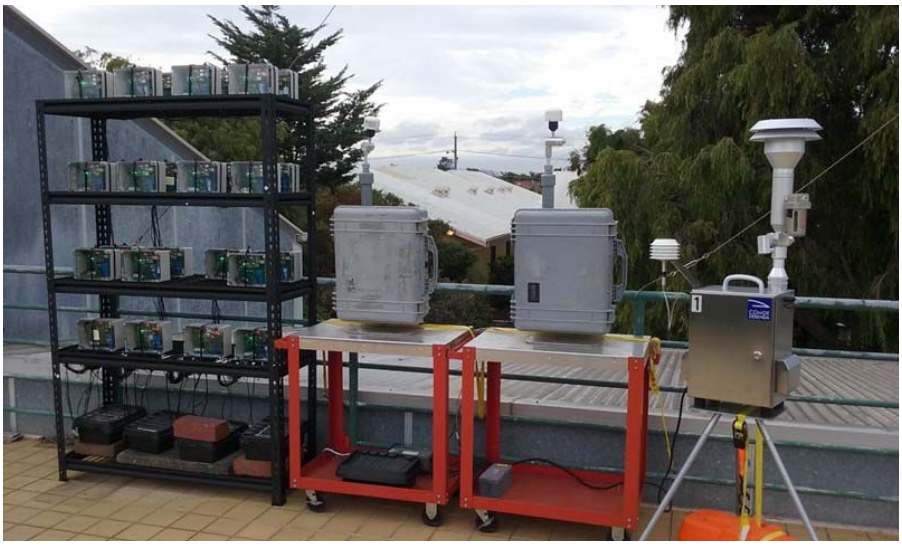

Figure S2. Set-up for intercomparison between SMOG units and E-samplers fitted with a $PM_{2.5}$ size selective inlet.

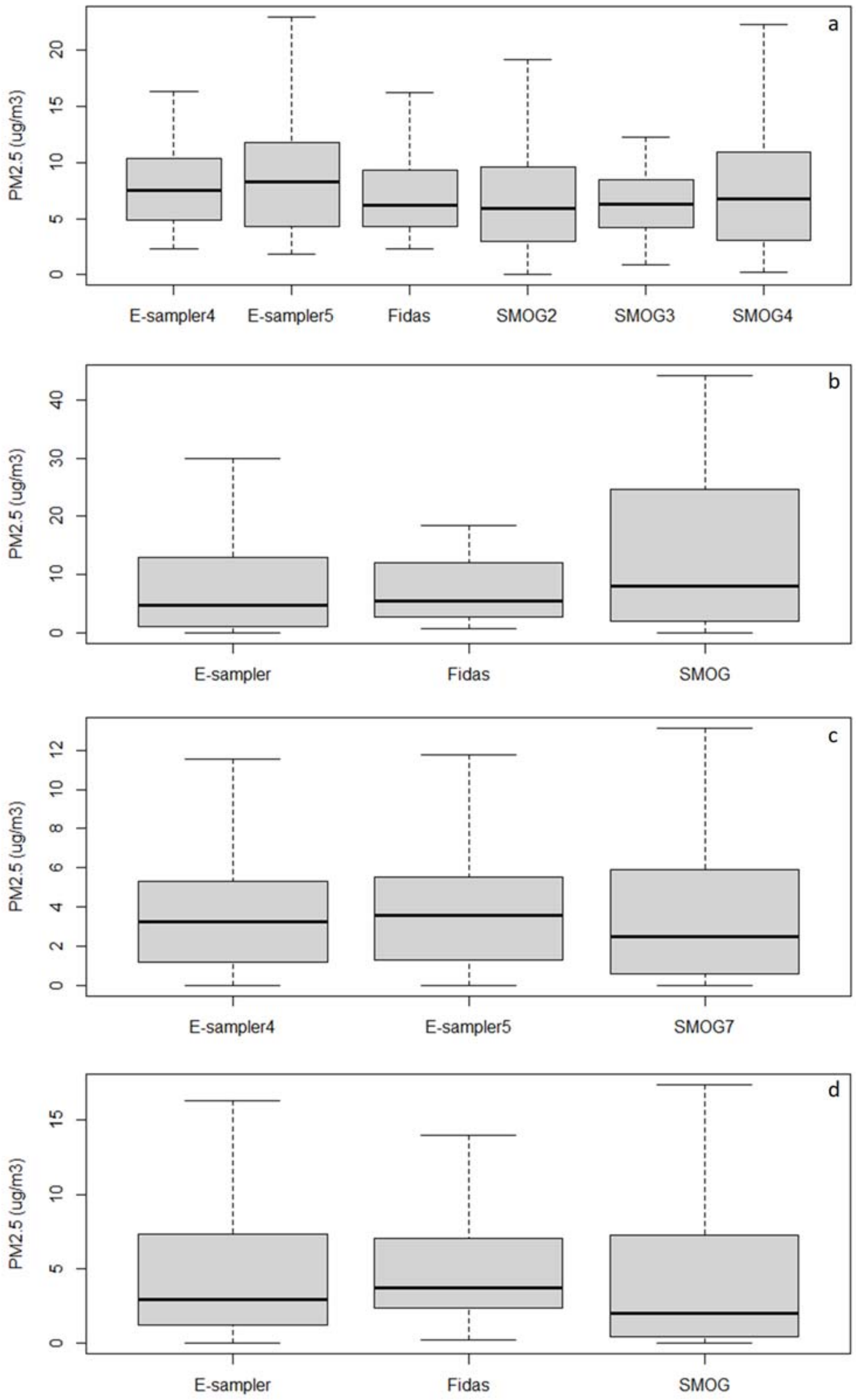

Figure S3. Boxplots for $PM_{2.5}$ hourly concentrations at (a) Aspendale Autumn, (b) Aspendale winter, (c) Rutherglen (Victoria) and (d) Alexandra (Victoria). Whiskers denote the 10th and 90th percentiles

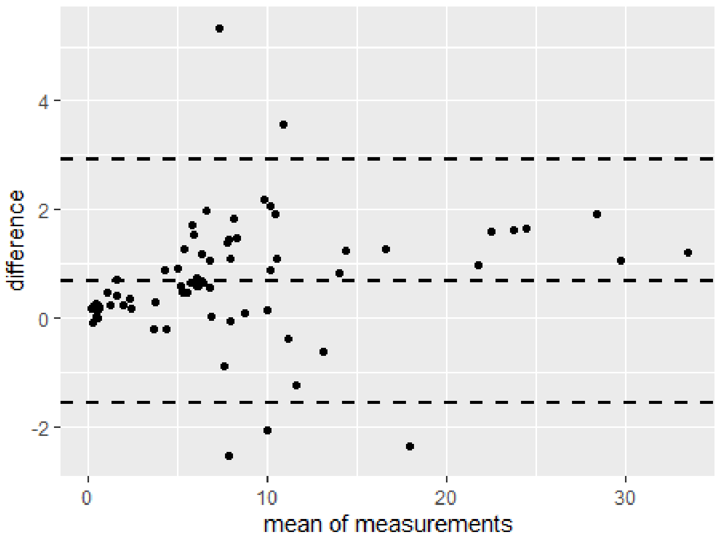

Figure S4. Bland-Altman plot of the agreement between $PM_{2.5}$ concentrations measured by two SMOG units during autumn at Aspendale.

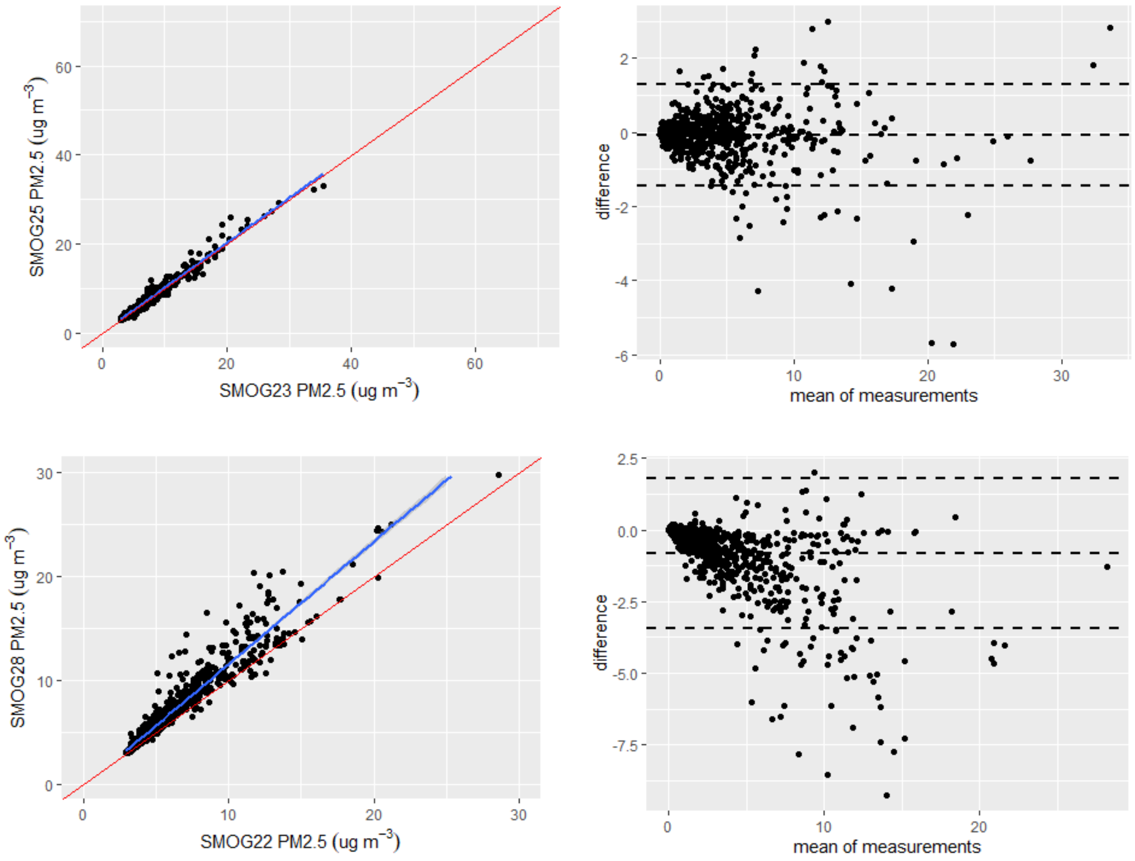

Figure S5. Scatter plot and Bland-Altman plots of the agreement between $PM_{2.5}$ concentrations measured by two SMOG units in Northeast Victoria.

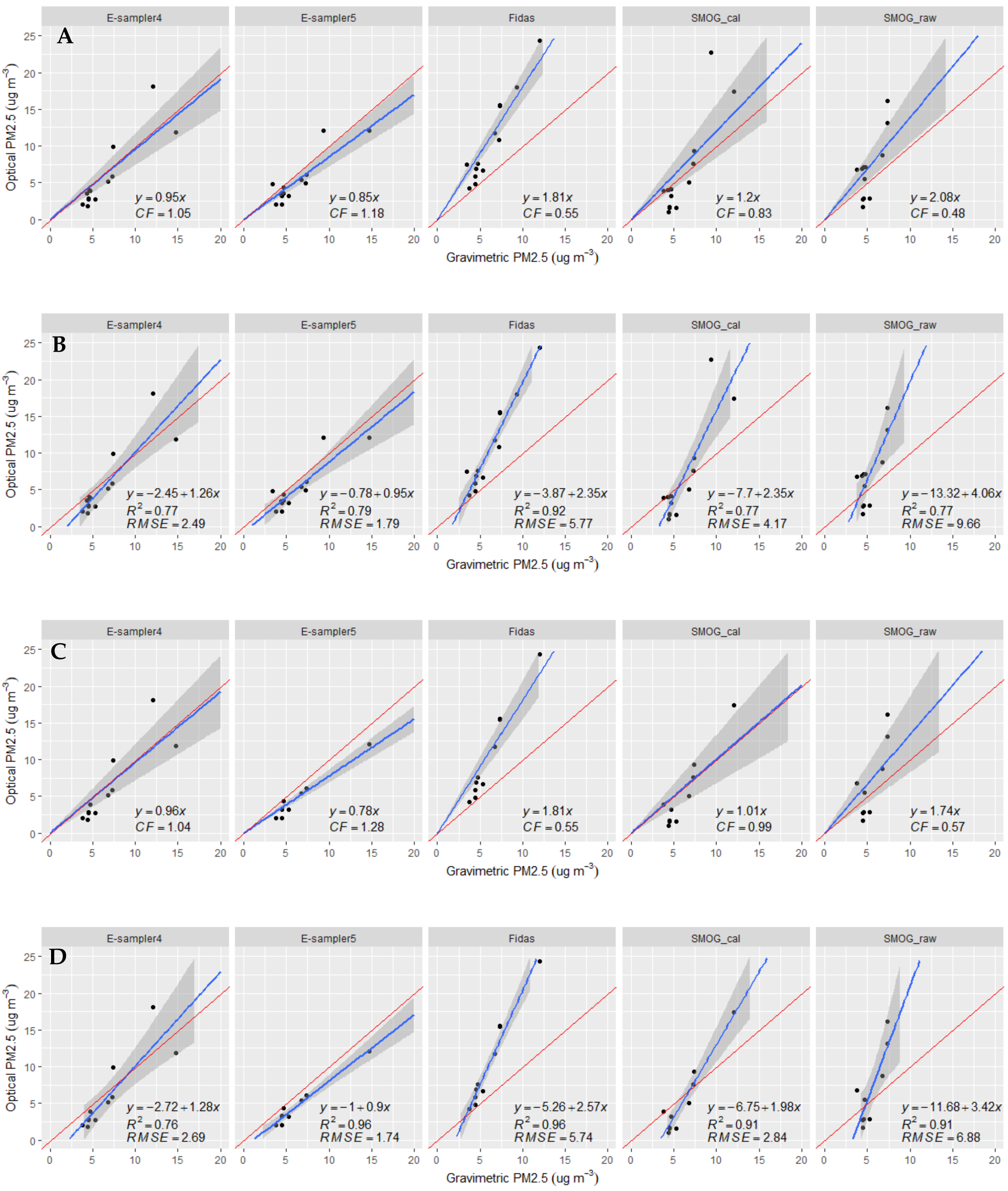

Figure S6. Linear regression analysis of optical measurements vs gravimetric mass measurements. A Regression with zero intercept for entire data set (CF represents the calibration factor = 1/slope); B Regression with non-zero intercept for entire data set; C Regression with zero intercept for Alexandra data set; D Regression with non-zero intercept for Alexandra data set. Blue lines show linear least-squares fit; red line represents 1:1 line.

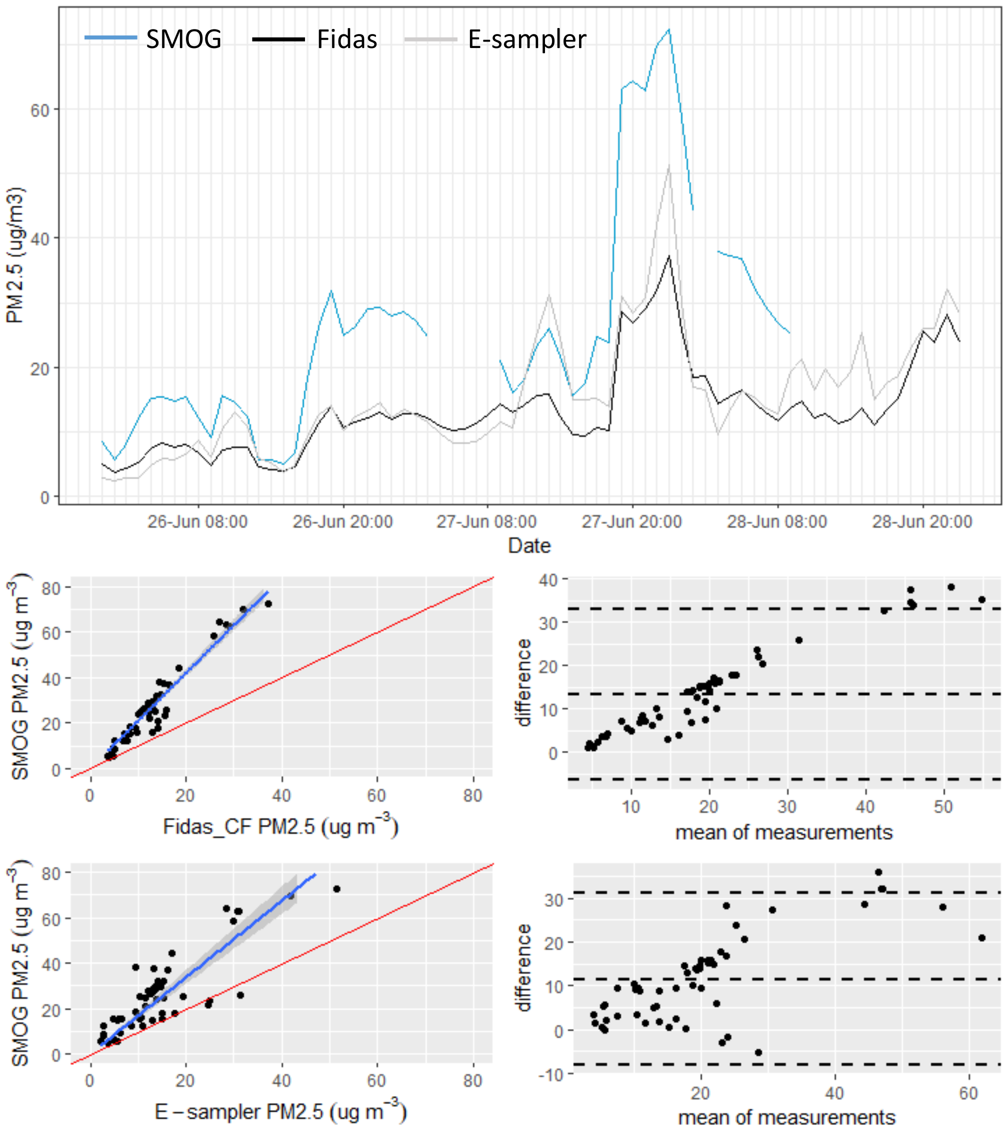

Figure S7. High particle event in winter at Aspendale.

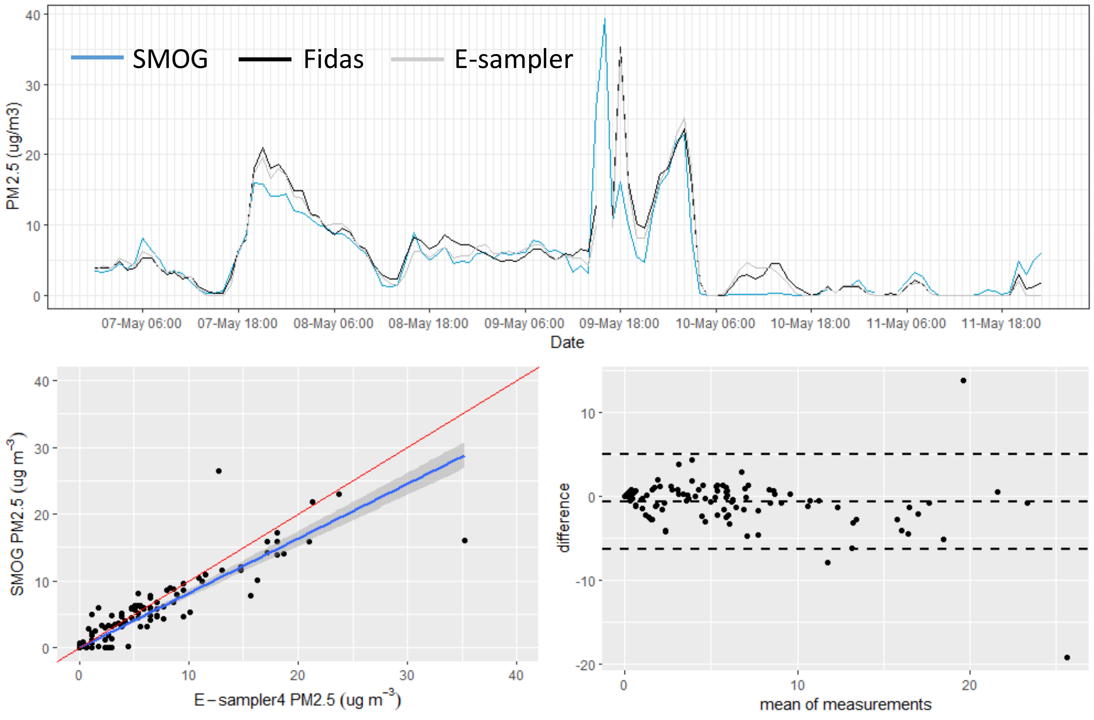

Figure S8. High particle event in autumn at Rutherglen.

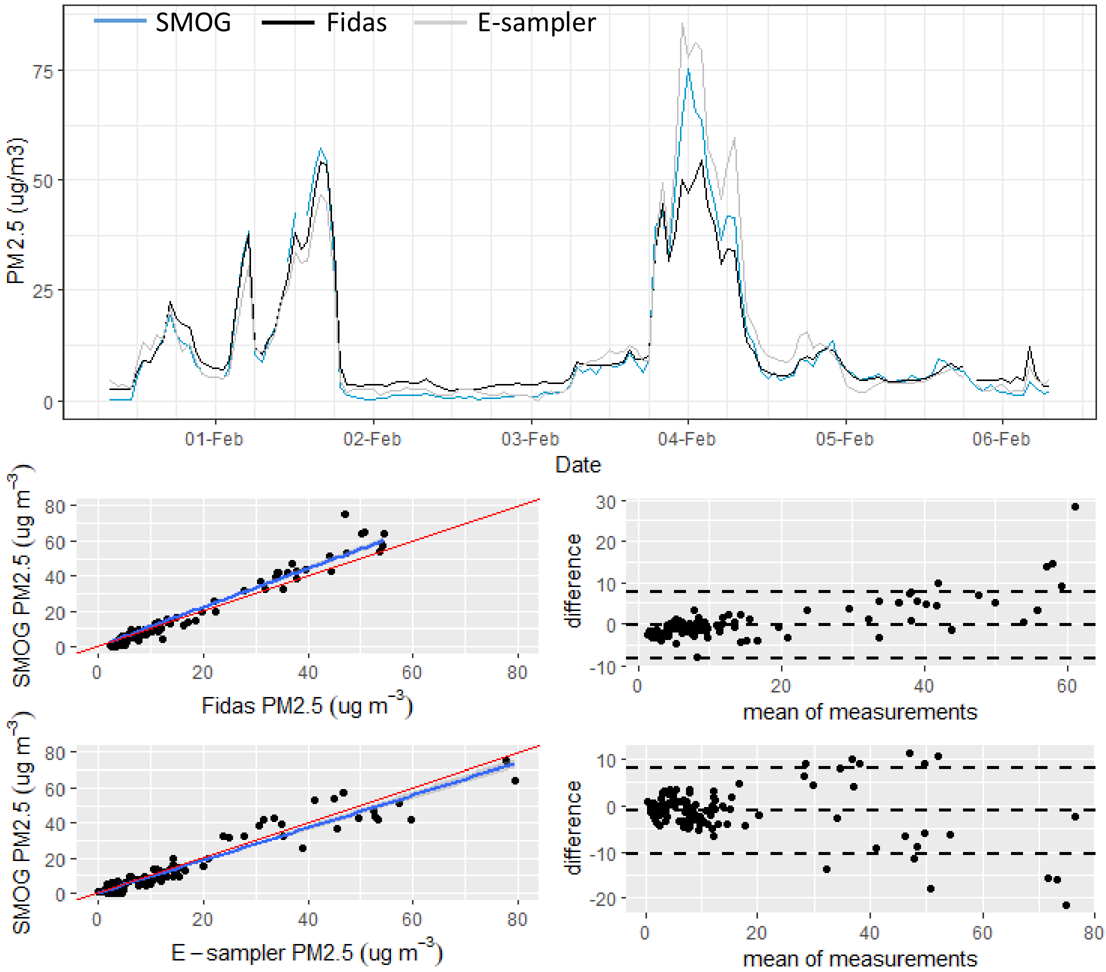

Figure S9. High particle event in summer at Alexandra

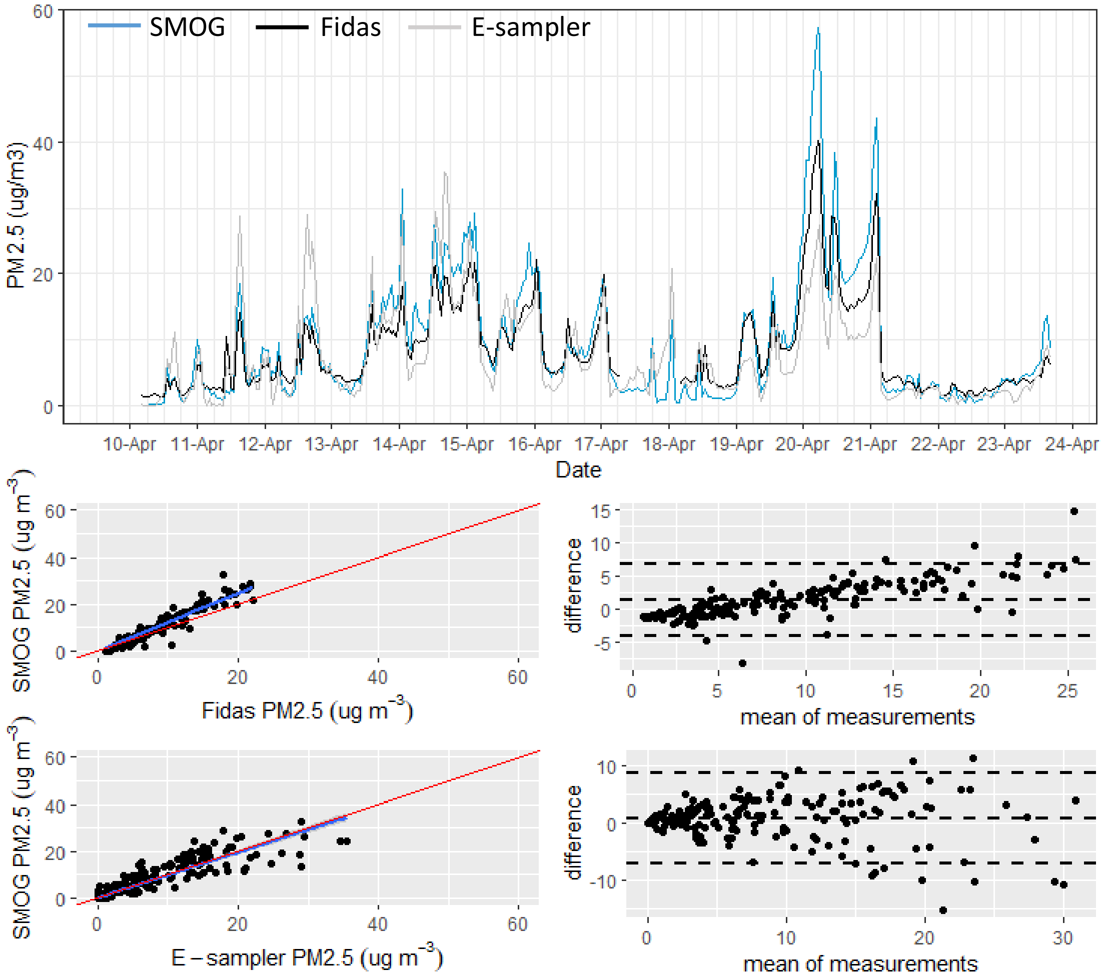

Figure S10. High particle event in autumn at Alexandra

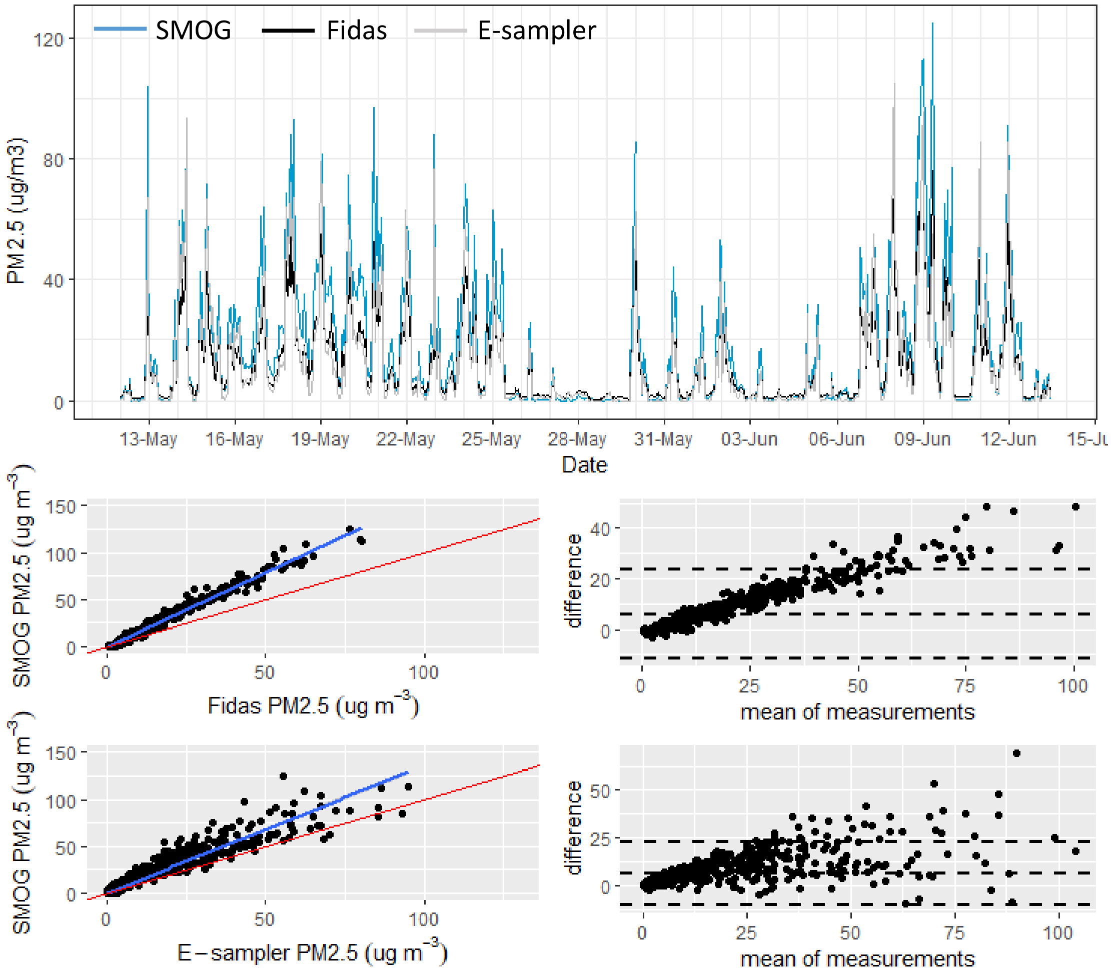

Figure S11. High particle event in winter at Alexandra
